# DNA replication machinery: Insights from *in vitro* single-molecule approaches

**DOI:** 10.1016/j.csbj.2021.04.013

**Published:** 2021-04-20

**Authors:** Rebeca Bocanegra, G.A. Ismael Plaza, Carlos R. Pulido, Borja Ibarra

**Affiliations:** IMDEA Nanociencia, Faraday 9, Campus Cantoblanco, 28049 Madrid, Spain

**Keywords:** DNA replication, Single-molecule, Force spectroscopy, Fluorescence spectroscopy, Replisome

## Abstract

The replisome is the multiprotein molecular machinery that replicates DNA. The replisome components work in precise coordination to unwind the double helix of the DNA and replicate the two strands simultaneously. The study of DNA replication using *in vitro* single-molecule approaches provides a novel quantitative understanding of the dynamics and mechanical principles that govern the operation of the replisome and its components. ‘Classical’ ensemble-averaging methods cannot obtain this information. Here we describe the main findings obtained with *in vitro* single-molecule methods on the performance of individual replisome components and reconstituted prokaryotic and eukaryotic replisomes. The emerging picture from these studies is that of stochastic, versatile and highly dynamic replisome machinery in which transient protein-protein and protein-DNA associations are responsible for robust DNA replication.

## Graphical abstract

**Figure:**
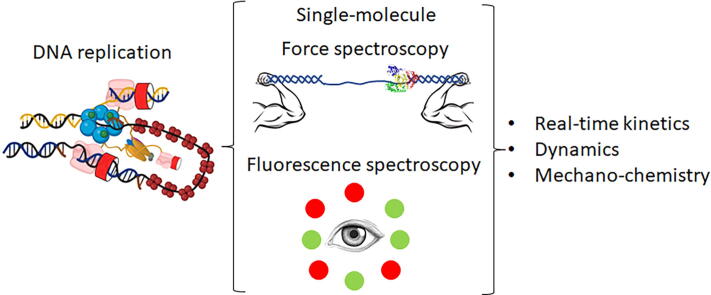

## 1 Introduction

**Fig. 1:**
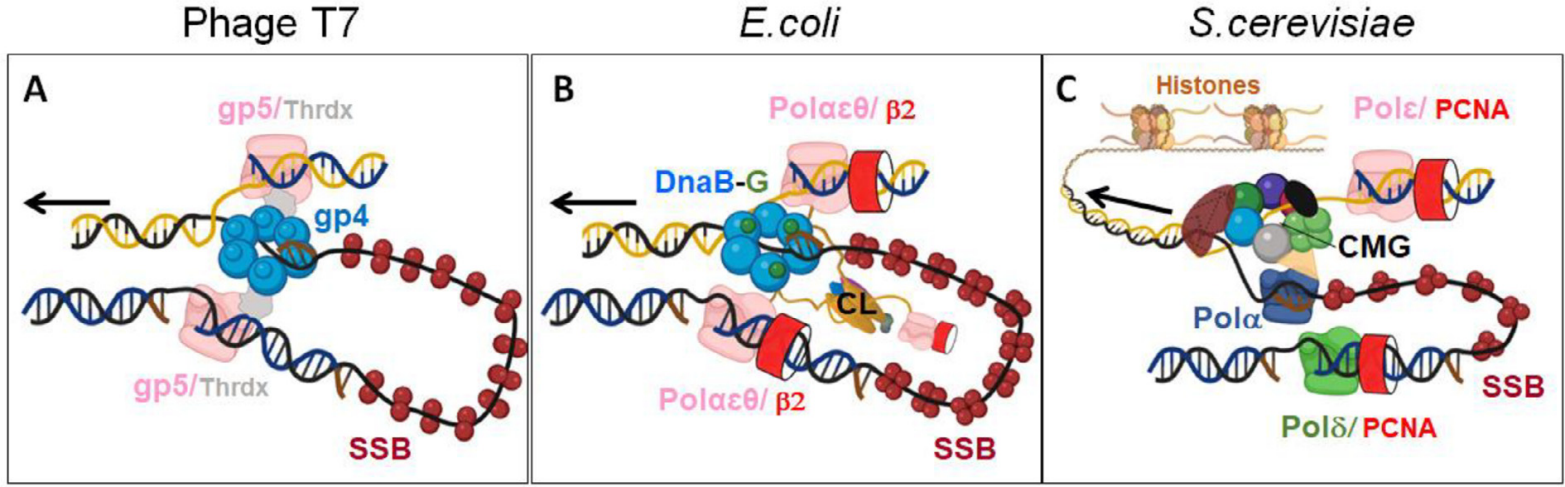
Schematic representations of replisomes of increasing complexity. For all figures arrows show the direction of the replication fork and leading strand (top) is depicted yellow and lagging strand (bottom) black. A) The bacteriophage T7 replisome is formed by 4 proteins: DNApol (gp5) and its processivity factor thioredoxin (Thrdx), the helicase-primase (gp4) and SSBs (gp2.5). The helicase (gp4) translocates in 5′–3′ direction on the lagging strand and synthesizes primers (brown) for the discontinuous synthesis of the lagging strand. Two or more DNApols (gp5) interact with the C-terminal tail of the helicase and replicate the two DNA strands. DNApols can also exchange with external DNApols at forks. The SSB gp2.5 covers exposed ssDNA regions and interacts with the DNApols and the helicase, regulating their activities. B) The *Escherichia coli* (*E.coli*) replisome is composed of at least 14 different protein subunits. The DnaB helicase translocates in 5′–3′ direction on the lagging strand, promotes strand separation, and interacts transiently with one or more DnaG primases for RNA priming (brown). The DNApol III holoenzyme is responsible for DNA synthesis and is made up of three subassemblies: (i) the αɛθ core polymerase complex that copy DNA, (ii) the β2 sliding clamp or processivity factor, and (iii) the seven-subunit clamp loader complex (CL) that loads β2 onto primer–template junctions and coordinates replication of the two strands. Up to three, readily exchangeable, core polymerase complexes bind to each fork. The coordinated synthesis of the two strands could be the outcome of the stochastic behavior of the DNApols at each strand. The SSB protein protects ssDNA and promotes helicase and DNApol activities. C) Up to 34 protein subunits built up the eukaryotic *S. cerevisiae* core replisome. The key components include: i) the 11-subunit heterohexameric CMG helicase that translocates on the leading strand in 3′–5′ direction, ii) three multi-subunit DNA polymerases: the leading-strand Pol ε, lagging-strand Pol δ, and Pol α-primase. Pol δ and Pol α are recycled to support the synthesis of multiple Okazaki fragments, iii) the replication factor C involved in attaching the processivity clamp (PCNA) to Pol δ, and iv) the RPA trimeric SSB protein. Numerous other proteins interact transiently with the eukaryotic replisome, some of which are known to be involved in checkpoint regulation or nucleosome handling, since in eukaryotes DNA is complexed to histones. (Adapted from [15]). (For interpretation of the references to colour in this figure legend, the reader is referred to the web version of this article.)

DNA replication is a fundamental process of life that has been a central focus of molecular biology. Only 5 years after the description of the double helical structure of the DNA in 1953 [1], the laboratory of Arthur Kornberg identified the first enzyme capable of synthesizing DNA, to which they referred as DNA polymerase (DNApol) [2], [3]. More than 60 years later, we are still gathering evidence to fully understand the robustness and beautiful sophistication of DNA replication and its regulation. The fundamental principles of DNA replication are surprisingly similar from simple viral systems up to the more complex organisms. The elegant experiment of Meselson and Stahl, a few years after the discovery of DNA structure, demonstrated that DNA is replicated in a semi-conservative fashion in which the two original DNA strands separate and each one serves as a template for a new DNA strand [4], Fig. 1. The antiparallel nature of DNA strands and the 5′-3′ polarity of DNApol force one of the strands to be synthesized continuously (leading strand) while the other (lagging strand) is synthesized discontinuously in shorter segments (Okazaki fragments), which are later joined together [5]. Despite these differences, the synthesis of the two strands is coupled, and is carried out by the same replication apparatus, called the replisome. The replisome is constituted by a sophisticated molecular machinery in which DNApols work in coordination with a plethora of other molecular motors (proteins that couple chemical energy to a mechanical task) and specialized proteins to unravel, synthesize, edit and move in one direction along mega-base-pair long genomes (Fig. 1). For example, the replisome of the bacterium *Escherichia coli* (*E.coli*, Fig. 1) is formed by at least 14 different protein subunits that synthetize DNA at rate up to 1,000 nucleotides per second with an accuracy of 1 wrong nucleotide incorporated every ~ 10^7^ nucleotide polymerized [6]. A copyist with comparable skills would copy Don Quixote's novel (~1,500 pages) in approximately 30 min without making a single typo. Over the last 60 years, biochemical, structural and genetic studies have been pivotal for identifying the components of the replication machineries in different organisms, and defining their functions and structures [5], [7], [8]. What is still missing is a detailed quantitative understanding of the dynamics and mechanical principles that underlie the operation of these molecular motors and their interactions with their partners at the replisome.

**Fig. 2:**
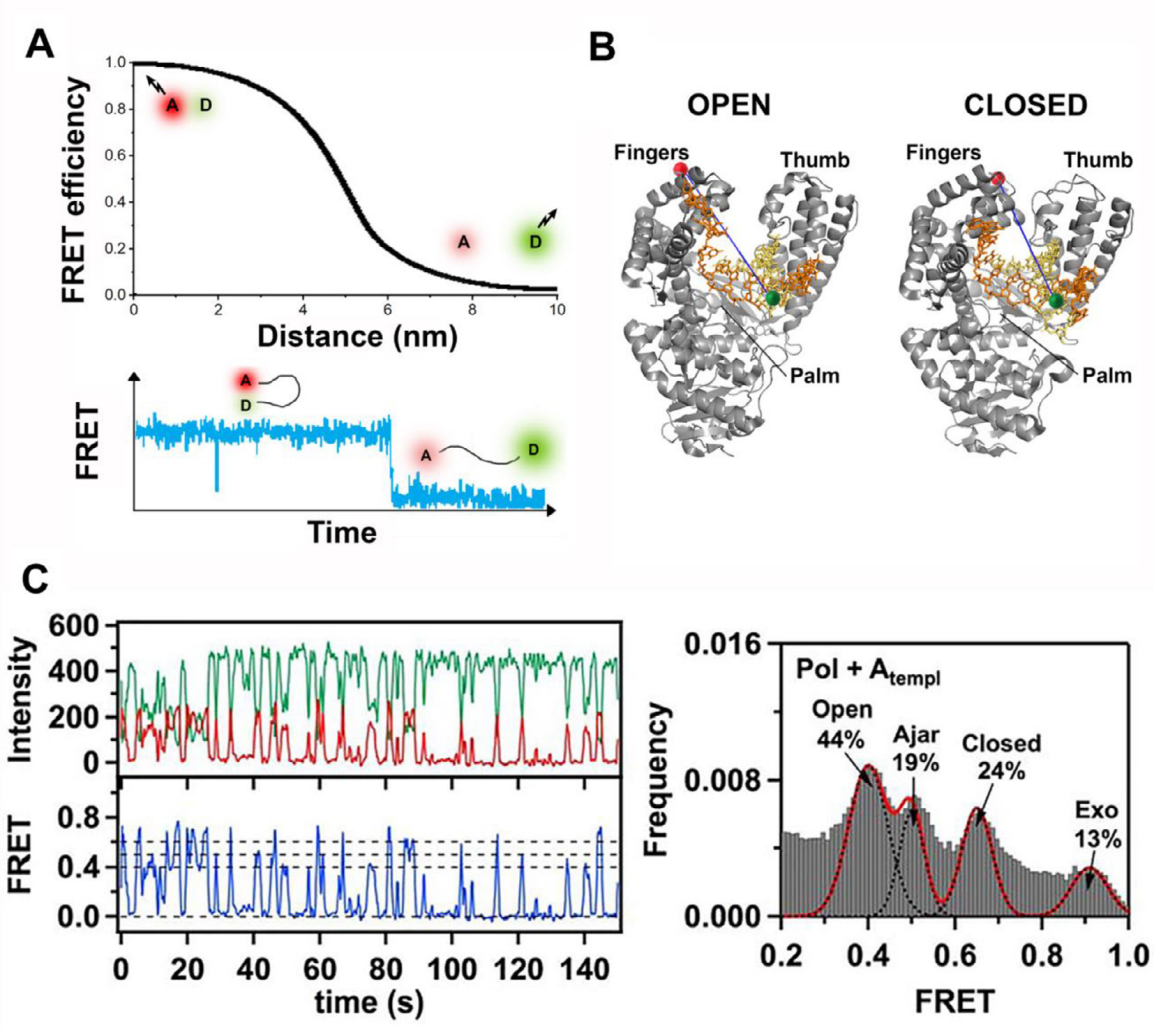
Single-molecule Förster resonance energy transfer (smFRET). A) smFRET is based on the non-radiative energy transfer between nearby located donor (green) and acceptor (red) fluorophores, which results in a decrease in the donor (green) and a concomitant increase in the acceptor (red) fluorescence signals. Monitoring the degree of energy transfer reports on the distance and dynamics of intra- and inter- molecular interactions on the sub–10 nm scale. Bottom panel shows a characteristic trace of FRET efficiency depending on donor and acceptor proximity (adapted from [9]). B) Schematic illustration of labeling strategy used to probe the finger-closing conformational change in Pol I Klenow fragment. The donor fluorophore (green) is attached to the primer DNA and the acceptor fluorophore (red) to the tip of the fingers subdomain. As the fingers pivot between the open and closed positions the distance between the two fluctuates, which induce changes in FRET signal. C) Left panel. Characteristic fluorescence intensity time traces (green donor and red acceptor), and smFRET efficiency trajectories (blue) for a DNApol-DNA complex labeled as in B. FRET efficiency histograms (right panel) show 4 major populations that the authors assigned to the open, ajar (intermediate) and closed conformations of the fingers subdomain, and a population of DNA bound at the distant exonuclease site (B and C panels are adapted from [41]). (For interpretation of the references to colour in this figure legend, the reader is referred to the web version of this article.)

**Fig. 3:**
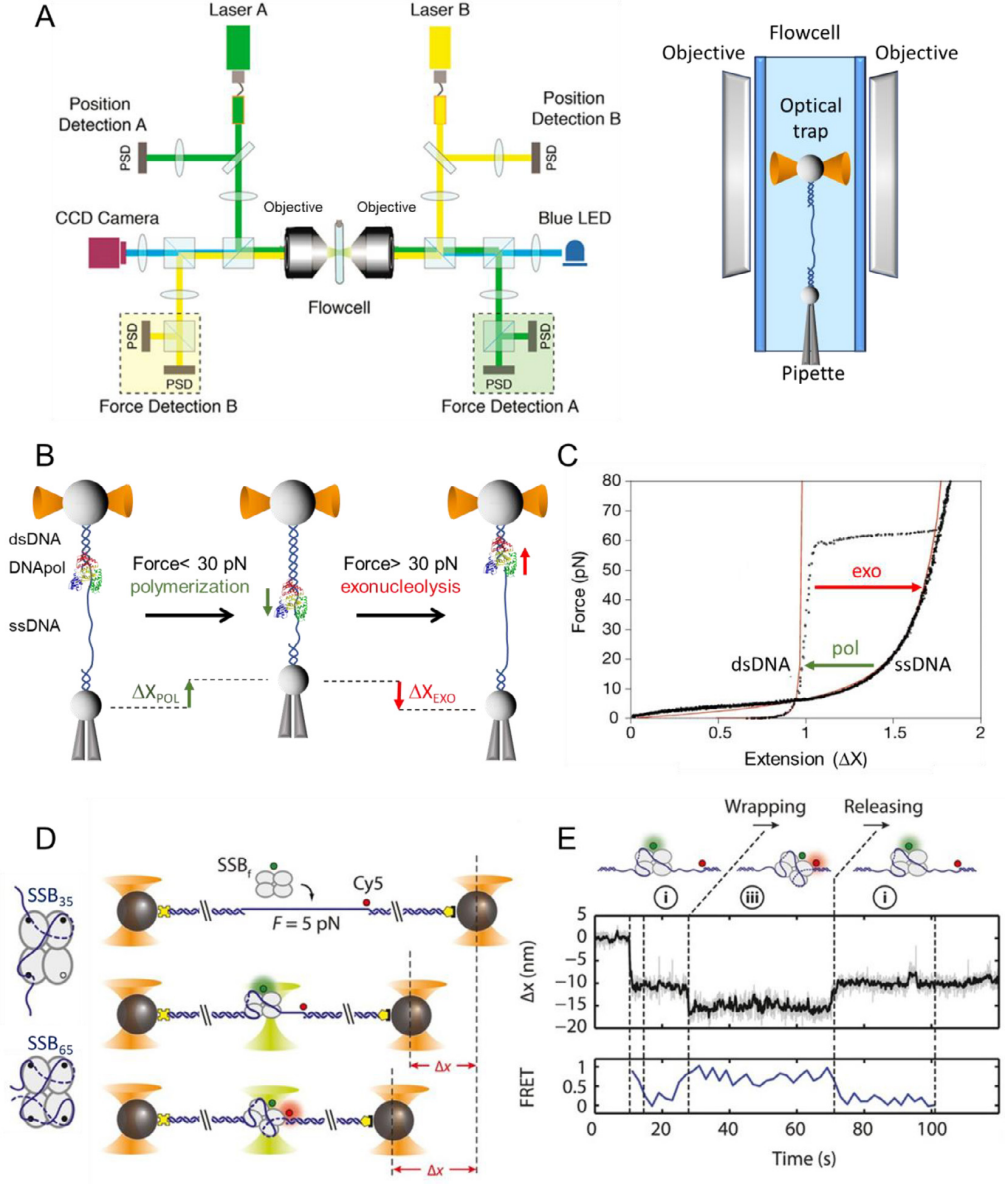
Optical tweezers and ‘Fleezers’. A): Diagram of a dual-beam optical tweezers setup. Two high numerical aperture objectives focus two counter-propagating 808 nm lasers, A (in green) and B (in yellow) inside a flow cell to form two optical traps. The position of each laser is controlled by piezo actuators. The two traps are superimposed in the same spatial position so that they function as one trap, effectively. To monitor the optical trap position beam-splitters divert a small percentage of the incoming light of each laser to position sensitive detectors (PSDs). The light leaving from each trap is sent to a different PSD to measure forces [100]. A CCD camera and a blue LED light (blue line) allow visualization of the interior of the flow cell (adapted from [101]). The panel on the right shows idealized lateral view of the flow cell showing a DNA molecule attached between two micron-sized polystyrene beads, one in the optical trap (orange cone) and the other on top of a micropipette. B) Experimental set-up to measure polymerization and exonucleolysis activities of individual DNApols with dual-beam optical tweezers [55]. A single DNA molecule containing a single-stranded gap is tethered to functionalized beads as in (A). At constant mechanical tension below 30 pN, the DNApol converts the single-stranded template (ssDNA) to double-stranded DNA (dsDNA). This activity is followed in real-time as a gradual shortening of the distance between the beads (Δx, green). Tension above 30–40 pN shifts the equilibrium towards the exonuclease activity, which is measured as a gradual increase in the distance between the beads (Δx, red). C) The force–extension curves of dsDNA and ssDNA can be described using polymer physics models (red lines) (reviewed in [102]). At constant force, the conversion from one polymer to the other by DNApol activities is captured as a change in extension D) Experimental set-up to measure the wrapping dynamics of *E.coli* SSB with a hybrid instrument that combines high-resolution optical tweezers with fluorescence detection (Fleezers, [103]). Polystyrene beads (grey) are held in separated optical traps (orange cones), tethered by a DNA molecule containing a short ssDNA region. The DNA is labeled with a FRET acceptor at the ss-dsDNA junction (red dot) and the SSB (tetramer) with the FRET donor (green dot). Fluorophores are excited by a ~ 500 nm laser (green cone). *E.coli* SSB binds to ssDNA and wraps either 35 or 65 nucleotides depending on the experimental conditions (as shown on the left diagram). ssDNA wrapping decreases the extension between the beads (Δx). E) Simultaneous measurement of tether extension (top) and FRET efficiency (bottom) enables determination of both the position of SSB along the tether and the amount of ssDNA wrapped (adapted from [104]). (For interpretation of the references to colour in this figure legend, the reader is referred to the web version of this article.)

**Fig. 4:**
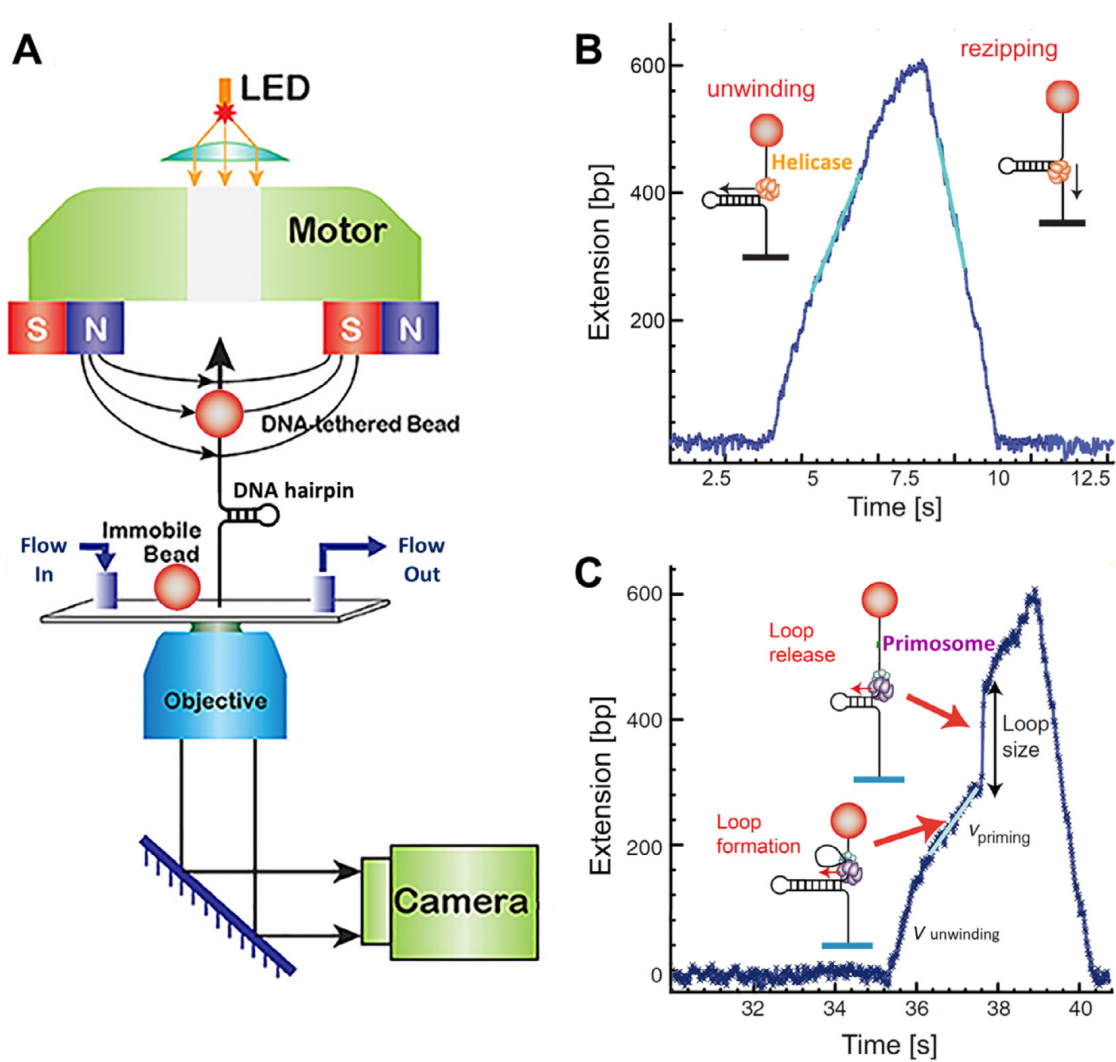
Magnetic tweezers. A) Diagram of a magnetic tweezers setup. A paramagnetic bead is tethered to the surface of a flow cell via a functionalized DNA molecule. Beads stuck directly to the surface are used as a reference for drift correction. Permanent magnets produce magnetic field that pulls the bead in the direction of the field gradient (arrows). The orientation of magnetic field exerts horizontal and/ or vertical magnetic forces to stretch force or twist the DNA molecule. A CCD camera is used to follow in real-time the motion of the tethered bead. The changes in DNA extension are recorded in real time by computer-assisted analysis of the bead image (adapted from [133]). B) Representative DNA unwinding trace of a single T4 helicase using magnetic tweezers. A DNA hairpin is tethered between the paramagnetic bead and the flow cell surface. At constant tension, the DNA unwinding activity of the helicase opens the hairpin, which results in an increase of the DNA molecule extension. Upon full unwinding, hairpin rezipping rate is limited by helicase translocation rate on ssDNA (adapted from [134]). C) Detection of the T4 primosome helicase and priming activities on DNA hairpins using magnetic tweezers. Experimental run showing: *i*) initial DNA unwinding rate by the T4 helicase (V_unwinding_), *ii*) apparent decrease in the unwinding rate due to priming loop formation (V_priming_), and *iii*) sudden extension increase due to loop release upon primer synthesis by the primase (Loop size). After hairpin unwinding, rezipping is limited by helicase translocation on ssDNA (adapted from [135]).

In the last two decades, the advent of *in vitro* single-molecule detection and manipulation methods has finally allowed researchers to begin to fill this gap (for review see [9], [10], [11], [12], [13], [14], [15], [16], [17]). These biophysical methods share the ability to follow the real-time trajectories of individual molecules with nanometer (<10 nm) and millisecond spatial–temporal resolutions [18], [19], [20], [21], [22], [23], [24], [25], [26]. In this way, rare or transient events of a reaction usually averaged out by ensemble techniques, such as pauses, backtrackings, and rate fluctuations become apparent, providing a dynamic picture of the reaction. Besides, *in vitro* single-molecule manipulation methods can be used to exert calibrated forces (0.1–100 piconewtons) on single biological molecules, and measure the forces that result from their operation. Direct access to these mechanical forces provides a unique opportunity to quantify the coupling of mechanical (motion) and chemical reactions that govern the operation of molecular motors [20]. Briefly, two main groups of techniques are being currently used to study DNA replication *in singulo*: fluorescence spectroscopy and force spectroscopy. Fluorescence-based single-molecule techniques allow the real-time observation of the trajectory of molecules labeled with single fluorophores, which are excited with a laser of the appropriate wavelength. There are two complementary fluorescence techniques that differ in their excitation and detection modalities, total internal reflection fluorescence (TIRF) and confocal [26]. When two different fluorophores are attached to the system of interest, single-molecule fluorescence resonance energy transfer (smFRET) can be measured between them [22], [24]. The fluorophores can be attached to different molecules to study their association and relative movements or alternatively, to different sites of the same molecule, allowing the measurement of conformational changes. In force spectroscopy methods [21], the dynamics of the protein acting on DNA are obtained by attaching the protein DNA-complex under study between a surface and a micron-size bead that is subjected to an external field. The nature of this external field, which dictates some of the main pros and cons of each technique, can be magnetic (magnetic tweezers), photonic (optical tweezers) or hydrodynamic (tethered particle techniques). The basic principles of operation of some of these techniques are briefly explained in the legends of Fig. 2, Fig. 3, Fig. 4, Fig. 6.

Here, we review the main highlights of recent *in vitro* single-molecule studies of some of the replisome’s main components; replicative DNApols, helicases, and single-stranded DNA binding proteins (SSBs) as well as recent developments in single-molecule research on fully or partially reconstituted replisomes.

## 2 Replicative DNA polymerases

Replicative DNA polymerases (DNApol) are the molecular motors responsible for synthesizing the new complementary strands of DNA. Helped by processivity factors, these enzymes use one strand of the DNA as a template and catalyze a processive stepwise addition of the corresponding complementary deoxynucleoside triphosphate (dNTP) on to the terminal 3′ end of the nascent DNA strand (primer).

The dNTP incorporation cycle involves large conformational changes of the DNApol subdomain referred to as the fingers, which pivots between ‘open and close’ positions in response to dNTP binding and hydrolysis reactions (Fig. 2) [27], [28], [29]. Structural and computational studies suggested that this conformational change could be coupled with translocation directly, pushing or pulling the DNApol to the next template position (power stroke models [30], [31]). In contrast, *in vitro* single-molecule nanopore [32], [33], [34], [35], [36], and optical tweezers [37] studies argued for a Brownian ratchet mechanism. According to this model, upon nucleotide incorporation, the DNApol diffuses freely between pre- and post-translocated states, and binding of the correct incoming dNTP stabilizes the post-translocated state [38]. The actual mechanism of translocation of DNA and RNA polymerases along nucleic acids is still a subject under investigation [39].

Accuracy during DNA replication is a must [40]. One of the two main factors contributing to fidelity is the ability of DNApols to select the dNTP complementary to the template strand. smFRET studies using the Klenow fragment of DNApol I as a model system followed the conformational dynamics of the fingers subdomain under various conditions and revealed the existence of previously unrecognized intermediates states within the open and closed transitions, Fig. 2 [41], [42], [43], [44], [45], [46], [47], [48]. These states may serve as kinetic checkpoints to discriminate against incorrect substrates during the dNTP incorporation cycle, conferring to the fingers conformational dynamics a novel role in replication fidelity. The second main factor contributing to fidelity is the capacity of DNApols to excise misincorporated incorporated nucleotides at the exonucleolytic active site (*Exo*). This site is separated by up to ~ 60 Å from the polymerization active site (*Pol*) and only binds single-stranded DNA [30], which imposes tight structural and kinetic requirements for efficient primer strand transfer. Single-molecule fluorescence [45], [49], [50], [51], [52], [53], and force spectroscopy, Fig. 3A and 3B, [54], [55], [56], [57], [58] studies on several replicative DNApols revealed that the primer transfer between the distant *Pol* and *Exo* sites, far from a one-step reaction, is a highly dynamic process that involves numerous conformational intermediate states along the proofreading pathway. These states may work as fidelity checkpoints essential to fine-tune the equilibrium between the *Pol* and *Exo* cycles required for robust but simultaneously faithful replication.

In addition, many DNApols present an intrinsic ability to unwind the DNA fork during replication. Ensemble measurements showed that this strand displacement activity is limited to a few nucleotides by the partition of the primer from the *Pol* to the *Exo* domains [59]. Magnetic and optical tweezers studies revealed that individual DNApols destabilize the fork’s next base pair with an average energy of 1–2 *k_B_T* per dNTP incorporated [60], [61], [62]. This energy is smaller than the average stability of the fork (~2.5 *k_B_T/ bp*) explaining why a stably closed fork junction slows down the polymerization rate, induces frequent pauses (as observed in smFRET studies too [63]), and shifts eventually the equilibrium towards the *Exo* conformation. These processes prevent excessive strand displacement activity by the lagging DNApol, which, as shown by *in vitro* ensemble studies, is detrimental for primer removal during Okazaki fragment maturation [64], [65]. During replication of the leading strand, engagement of the helicase (and presumably SSBs) with the displaced strand would help decrease the energy barrier for DNA unwinding, preventing the *Pol-Exo* partition. Under these conditions, both enzymes would coordinate their DNA unwinding properties to promote processive DNA replication [66], [67].

## 3 Replicative DNA helicases

Replicative helicases form hexameric rings that utilize energy derived from binding and hydrolysis of nucleoside triphosphates (NTPs) to translocate along ssDNA and partially destabilize the fork junction to facilitate DNA unwinding [68], [69], Fig. 4. Interestingly, eukaryotic (and archaeal) helicases form hetero-hexameric rings that encircle the leading strand in its central channel and translocate in the 3′−5′ direction. In contrast, their prokaryotic counterparts form homo-hexameric rings that encircle the lagging strand and translocate in the 5′−3′ direction [70]. In both cases, unwinding of the fork is promoted by steric exclusion of the non-circled strand from the central channel [68], [70], [71], [72]. In addition to DNA unwinding, hexameric helicases play a fundamental role as one of the central organizing centers of replisomes.

*In vitro* single-molecule studies have contributed significantly to decipher the operation of these molecular motors [73]. Together with ensemble studies, *in singulo* research showed that eukaryotic and archaeal helicases load onto duplex replication origin DNA as double-hexamers in a sequential manner [74], [75], [76], [77]. Then, a set of 'firing factors' are required to convert each double hexamer into two active helicases competent for DNA unwinding and replisome progression [78], [79], [80], [81], [82], [83]. Although the translocation mechanism of eukaryotic-type helicases is still under debate, magnetic tweezers studies suggested that the eukaryotic CMG translocate and unwinds DNA via an random walk biased by ATP binding/hydrolysis with a high propensity to pause in the absence of accessory factors [84]. For prokaryotic helicases, single-molecule studies (together with biochemical and structural measurements) supported a sequential hand-over-hand translocation mechanism with an overall kinetic step size of 1 bp/NTP, which may depend on the sequence context [85], [86], [87], [88].

Overall, single-molecule studies revealed that the real-time kinetics of replicative helicases is frequently interrupted by pauses and slipping events, and strand separation is the rate-limiting step of their mechano-chemical cycle [87], [89], [90], [91], [92], [93], [94]. The poor unwinding ‘activeness’ of replicative helicases would avoid replisome uncoupling upon DNApol stalling [95], and suggest that their activity would be strongly regulated within the replisome to achieve rapid and processive replication. In fact, single-molecule and bulk studies have shown that slippage and pause events decrease and DNA unwinding rates increase when the helicase works at the fork in coordination with DNApol, primases [66], [67], [86], [96], [97], [98], [99] and/or SSB proteins (see below).

## 4 Single-stranded DNA binding proteins (SSBs)

SSB proteins are essential for the replisome’s proper operation and play pivotal roles during genome maintenance (for review [105], [106]). During DNA replication, SSBs bind to the lagging strand with high affinity in a sequence-independent manner and constitute the nucleo-protein complex upon which other components of the replisome work. Many SSB contain several Oligosaccharide Binding domains (OB-folds), allowing them to bind a variable number of nucleotides *in vitro* (review in [107]). These different binding modes may be used selectively in different DNA maintenance processes [108].

Single-molecule studies have revealed new information about the equilibrium constants and energetics of the binding of several prokaryotic and eukaryotic SSBs to individual ssDNA molecules [109], [110], [111], [112], [113], [114], [115], [116], [117], [118], [119], [120]. One of the most extensively studied SSB proteins at the single-molecule level is the homo-tetrameric SSB of *E. coli* (EcoSSB). Depending on the ionic conditions and SSB density on ssDNA, EcoSSB wraps *in vitro* ~ 17, 35 or (56)65 nucleotides/ tetramer [121]. smFRET and force spectroscopy measurements uncovered a highly dynamic binding of EcoSSB to ssDNA, in which the major binding modes can interchange reversibly in discrete steps [104], [122] and, individual EcoSSB tetramers can diffuse along ssDNA by a reptation mechanism [123], [124] while in different binding modes [104], Fig. 3D and 3E. These results explained how EcoSSB could be redistributed along ssDNA by genome maintenance proteins and remain tightly bound to ssDNA. Diffusion along ssDNA has also been reported at the single-molecule level for other SSB proteins [109], [120], [125]. Also, single-molecule fluorescence and force spectroscopy studies showed that on long ssDNA segments, EcoSSB can interact with distant intramolecular sites [126] and reposition itself via long-range intersegment transfer [127]. Single-molecule imaging of labeled EcoSSBs showed that intersegment transfer also occurs during DNA replication *in vitro and vivo* and SSB recycling for multiple Okazaki fragments would depend on the concentration of competing SSBs in solution [128]. Concentration dependent exchange was also reported at the single-molecule level for the eukaryotic RPA SSB protein [110].

*In vitro* single-molecule studies also revealed that at the replication fork SSBs stimulate the average rates and processivity of the lagging and leading strand DNApols as well as those of the replicative helicases, by establishing functional and/or physical interactions with these molecular motors [80], [129], [130], [131]. Simultaneously, the gradual release of the lagging strand during DNA replication has been shown to select the binding mode of the human mitochondrial SSB [132], highlighting the reciprocal interactions between the replisome components at the fork.

## 5 Replication machineries: Replisomes

The composition of the replisome varies among different organisms extensively. However, the structure and physical–chemical properties of the DNA impose basic operating principles to replisomes, Fig. 1. Next, we will summarize the main findings of single-molecule studies on the operation of model prokaryotic and eukaryotic replisomes.

As the first step for DNA replication, the replisome components assemble at the replication origin. smFRET studies revealed the ATP-dependent assembly pathway of the T4 replisome. Interestingly, while the T4 DNApol could use multiple pathways to load on the leading strand [136], the primosome (helicase and primase) assembles into the lagging strand in a single and orderly fashion [137], [138]. Upon helicase loading, 1–3 primase molecules bind to the helicase hexamer, which in turn, stabilizes the complex on the DNA fork and stimulates helicase activity [138], [139], [140]. Simultaneously, helicase loading turns on the activity of the leading strand DNApol holoenzyme [141]. Overall, these results showed a finely-tuned orchestration between replisome components to ensure a proper replisome assembly on to the DNA.

**Fig. 5:**
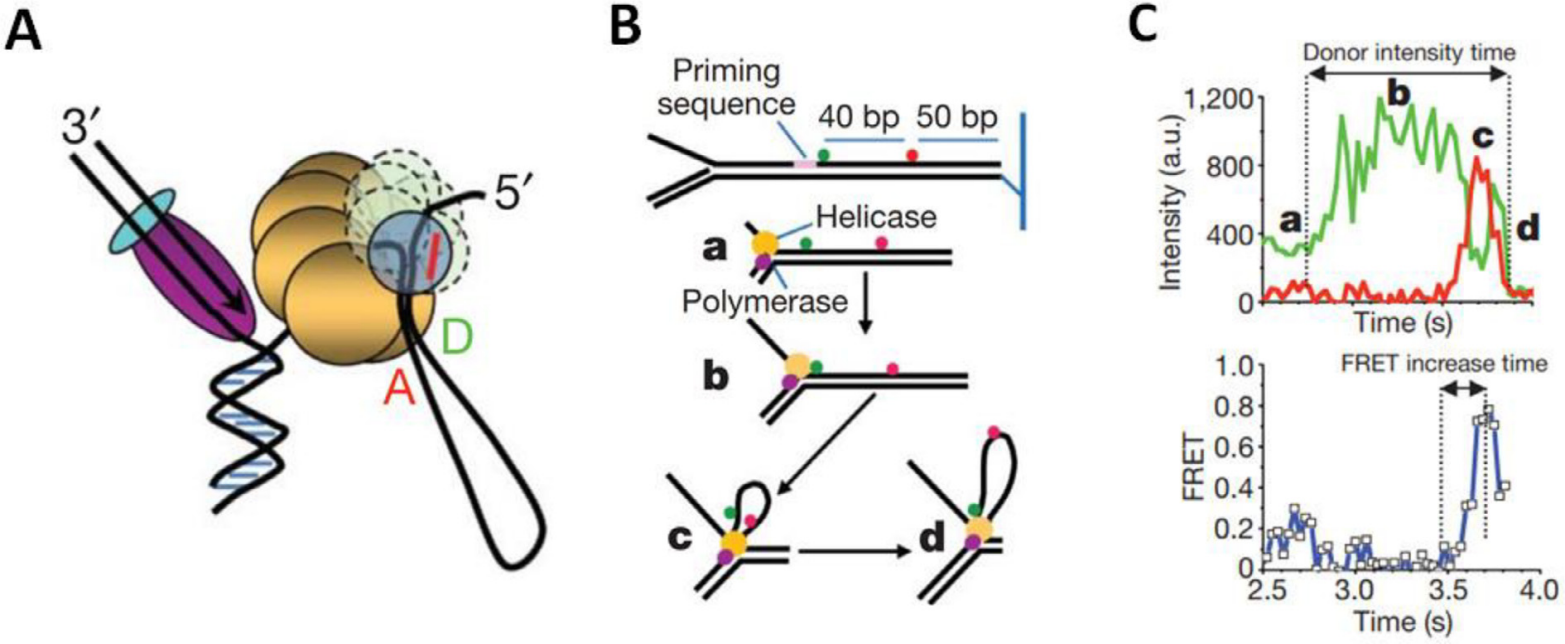
smFRET detection of priming loop formation by the T7 replisome. A) Diagram showing a priming loop during the activity of a partially reconstituted T7 replisome. In T7, helicase and primase activities are carried out by the same polypeptide (gp4). During primer synthesis (red line), the excess DNA unwound by the helicase activity loops out allowing the primase-DNA interaction to stay intact as leading strand synthesis proceeds. Red A and green D, represent DNA bound acceptor and donor fluorophores, respectively, used to detect primosome activity. B) Schematic representation of fluorescently labelled DNA fork to investigate priming loop formation by smFRET. Red and green dots show the location of the acceptor and donor fluorophores, respectively, with respect to the priming sequence (pink). C) smFRET unwinding assays show: a) Before DNA unwinding the distance between the two fluorophores prevents FRET (bottom plot). b) As the T7 replisome unwinds the dsDNA, the donor shows an increase in intensity (green trace) due to protein-induced fluorescence enhancement. c) When the replisome reaches the priming sequence, the primase domain engages the lagging strand at this position causing the acceptor (red trace) to come close to the donor, as DNA unwinding continues. This event is detected as an increase in FRET. d) As the priming loop grows in size the donor and acceptor move apart, this was detected as a decrease in FRET. Adapted from [145]. (For interpretation of the references to colour in this figure legend, the reader is referred to the web version of this article.)

The antiparallel nature of the lagging and leading DNA strands forces a precise series of highly coordinated events within the replisome to ensure the synchronized synthesis of the two strands. On the one hand, leading and lagging strand DNApols move in opposite directions (Fig. 1). In prokaryotes, ensemble studies showed that this problem is solved by forming of a ‘trombone loop’ in the lagging strand to reorient the lagging-strand DNApol to advance in parallel with its leading-strand counterpart, Fig. 1 [142], [143]. Single-molecule fluorescence and flow stretching assays with the reconstituted T7 replisome followed the dynamics of ‘trombone loop’ formation and revealed that two events ensure the timely release of loops: the primer synthesis and the actual completion of the Okazaki fragment [144]. On the other hand, the primosome faces a similar directionality problem; the primase (usually associated with the helicase) makes primers opposite to helicase movement (Fig. 1). Single-molecule studies showed that T7 and T4 replisome components overcame this problem by the transient formation of a 'priming loop' between the helicase and the primase [135], [145], Fig. 4C and Fig. 5. This mechanism keeps the primer in physical proximity to the replication complex and ensures hand-off to the lagging-strand polymerase without transiently blocking the replisome advance [145]. The T4 study also showed that one of the primase subunits can dissociate from the primosome complex to remain with the newly synthesized primer [135].

The discontinuous synthesis of the lagging strand also requires either replacement or recycling of the lagging DNApol to the next Okazaki fragment*. In vitro* single-molecule fluorescence studies showed that the T7 replisome addresses this issue by associating several DNApols with the replisome [96], [146], which are exchanged continuously at the lagging strand at a frequency similar to that of Okazaki fragment synthesis [147]. In addition, some of the lagging strand DNApols can be released from the replisome to complete Okazaki fragment synthesis behind and independent of the replication complex [148]. Similarly, ensemble and *in vivo* studies revealed that the *E.coli* replisome also contains more than two DNApols; Up to three DNApol (Pol III) cores could work in coordination and exchange at the fork while remaining attached at the replisome [149], [150], Fig. 1. A tripolymerase replisome has been shown to present functional advantages such as increased processivity and increased efficiency in lagging-strand synthesis [151]. In addition, *in vitro and in vivo* single molecule fluorescence experiments on T7 [146], [147] and E. coli [152], [153] replication systems showed that DNApols associated with the replisome can also be exchanged with other DNApols in solution in a concentration dependent manner. DNApol exchange was also demonstrated in ensembles studies for the bacteriophage T4 [136], [154]. In addition to DNApols, dynamic exchange has been reported also between different types of polymerases [155], [156], [157] and for other components of the replisomes [158]. These observations depict the replisome as highly dynamic molecular entity. The dynamic exchange of polymerases at the fork, by molecules already associated with the replisome or by proteins in solution, promotes the processivity of the replication complex and may allow the recruitment of factors necessary to correct lesions, overcome protein barriers in the DNA template, or replace a damaged polymerase without dismantling of the replisome structure [159].

**Fig. 6:**
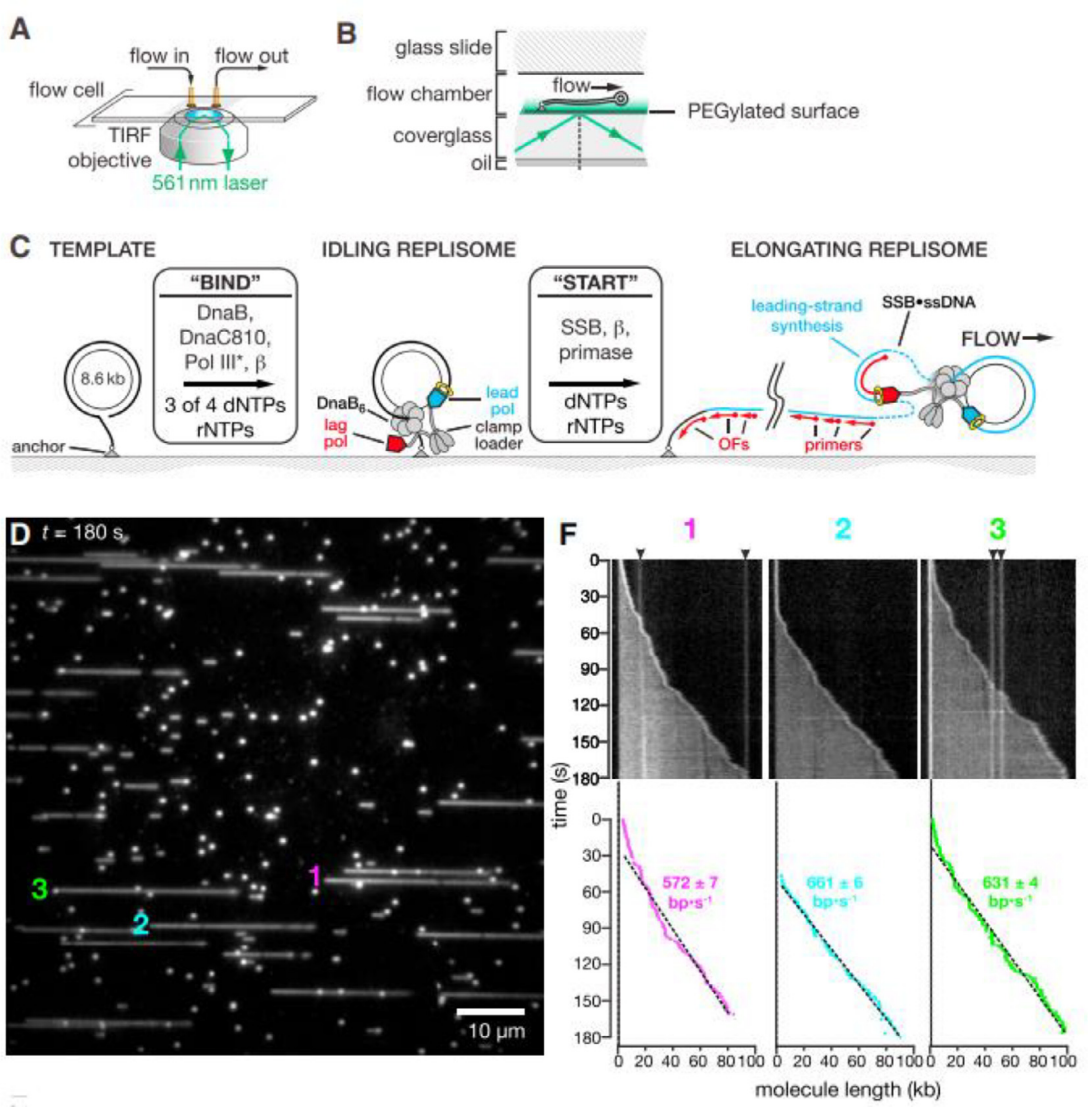
Single-molecule TIRF assays to visualize leading- and lagging strand synthesis by the *E.coli* replisome. A) Schematic of a single-molecule TIRF microscope and flow-cell. TIRF microscopy use evanescent waves to excite only those molecules located within ~100 nm of the surface, substantially reducing the background fluorescence. B) Side-on view of the flow cell, showing surface-attached DNA, flow direction, the excitation beam (561 nm, green lines) and the evanescent wave range (green). C) Diagram of the rolling-circle assay to detect single-turnover replisome progressions. The template was adsorbed onto a cover-glass via biotin-streptavidin interaction. Upon assembly of a pre-initiation complex (BIND), replication was initiated (START) by introducing primase, clamp, SSB in the presence of all four dNTPs and rNTPs. D) dsDNA extension can be followed in real-time by stretching under flow (from left to right) in the presence of SYTOX Orange. The figure shows a representative field in which several circular template molecules (small foci at the start of reaction) are replicated to yield long products. F) Kymographs of three actively extending molecules (from D) showing the length of the replication product as a function of time. Bottom, linear fits to trajectories yield average rates of fork movement (magenta, cyan, and green traces). Adapted from [95]. (For interpretation of the references to colour in this figure legend, the reader is referred to the web version of this article.)

Overall, the discontinuous synthesis of the lagging strand implies a series of ‘slow’ steps, which are not required for the continuous synthesis of the leading strand. Single-molecule studies suggested two alternative mechanisms to explain how the discontinuous lagging strand synthesis would keep pace with that of the leading strand: 1) the lagging strand synthesis or primase activity would halt the advance of the leading strand transiently [99], [130], [160], [161]. 2) The lagging-strand DNApol synthesizes DNA faster than the leading strand polymerase [145]. Interestingly, the prevailing deterministic view of a coordinated synthesis of the two strands was challenged recently by *in vitro* single-molecule fluorescence (TIRF) studies with the reconstituted *E.coli* replisome [95]. This study presented clear evidence showing that instead of a deterministic coupling, the two strands could replicate autonomously, Fig. 6. The observed coordination would be the outcome of the stochastic behavior of the DNApols at each strand, which start, stop, and move at variable rates.

**Fig. 7:**
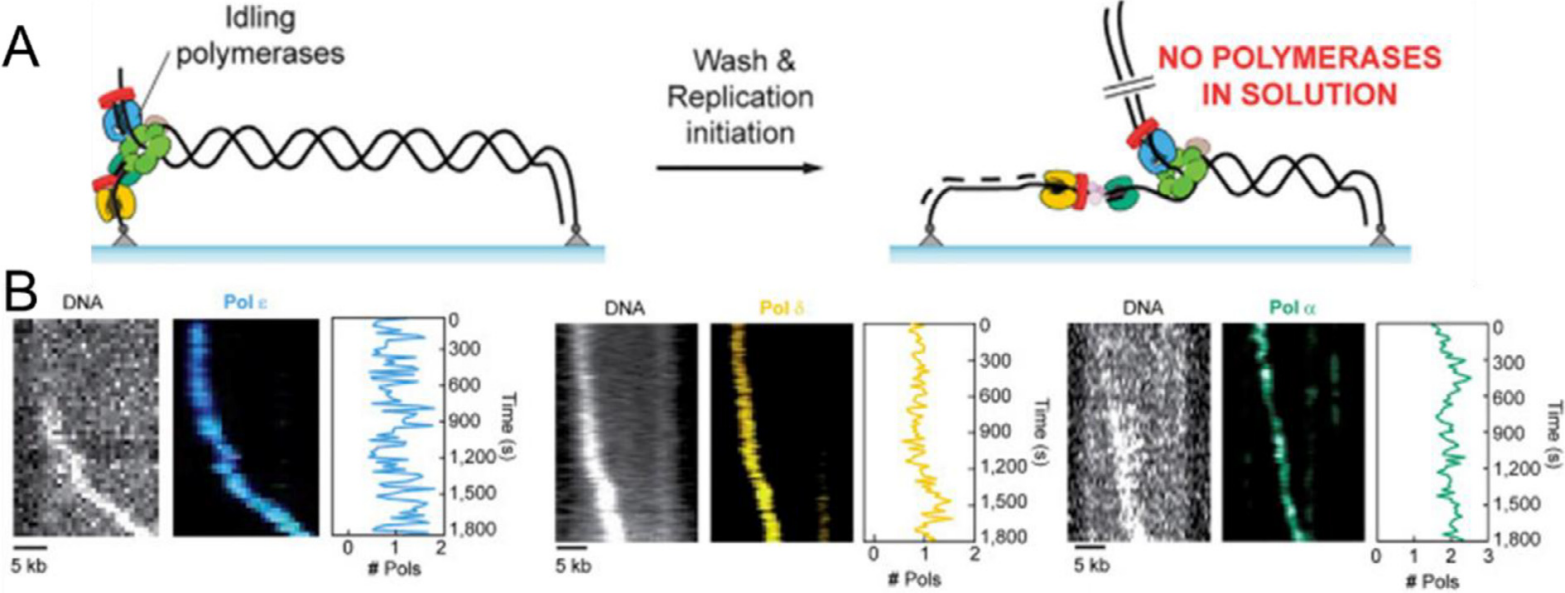
Multicolor single-molecule TIRF assays to visualize simultaneously DNA synthesis and protein dynamics of the *S. cerevisiae* replisome. A) Schematic representation of the pre-assembly replication assay. A DNA molecule containing a premade replication fork at one end is attached at both ends to the surface of the flow cell of a TIRF microscope. Upon preassembly of the replisome, the flow cell is washed to remove the excess of DNApols and other replisome components and replication is initiated. B) Kymographs showing the advance of the replication fork and the stability and stoichiometry of eukaryotic DNApols. DNA was stained with SYTOX orange and Pol ε (blue), Pol δ (yellow), and Pol α-primase (green) were labeled fluorescently. As DNA synthesis proceeds, the leading strand appears as a diffraction-limited spot that moves along the template in one direction (left). All three DNA polymerases co-localize with the leading-strand spot during replication of thousands of nucleotides (center). This observation is consistent with a stable interaction of the DNApols with the replisome. The stoichiometry of each DNApol (right) was obtained by dividing the intensity at the fork by the intensity of a single polymerase. Adapted from [162]. (For interpretation of the references to colour in this figure legend, the reader is referred to the web version of this article.)

The emerging picture coming out from *in vitro* single-molecule studies in prokaryotes is that of a stochastic, dynamic replisome in which protein–protein and protein-DNA associations are continually broken and reformed. Interestingly, recent *in vitro* [162], [163] and *in vivo* [164] single-molecule studies of *S. cerevisiae* replisome operation showed that in eukaryotes, the lagging strand DNApols, and other subunits of the replisome, also present dynamic exchange but associate more stably with the replisome than their prokaryotic counterparts, Fig. 7. These pioneer works point to relevant differences between the operational dynamics of prokaryotic and eukaryotic replisomes and forecast exciting discoveries in the near future.

## 6 Summary and outlook

In the last 60 years, the combination of biochemical, structural, genetic, and more recently, single-molecule approaches has provided a solid understanding of the molecular mechanisms underlying the complex choreography of the replisome components during replication of the double helix of the DNA. We have identified the leading dancers, their looks and their roles. We have also begun to realize the stochastic nature and the high adaptability of the replisome machinery, which changes its composition and operation mode continuously. This property would play an essential role in coping with constraints associated with the various stages of DNA replication and would ensure robust replication under varying conditions.

However, a complete understanding of the replisome operation will require defining the basic mechanistic, kinetic and dynamic processes that rule its operation at the molecular level and how these processes respond to external variables. Bottom-up *in vitro* single-molecule approaches, moving gradually from the study of individual components to increasingly complex replisomes, will help to set the path to addressing these challenges in combination with biochemical, structural and genetic advances. The assembly of robust reconstituted replisomes *in singulo* will be pivotal to exploring the effects of post-translational modifications, DNA roadblocks, DNA bound proteins, and disease-related mutations on the replisome operational dynamics.

*In vitro* single-molecule research will have to surmount several challenges and limitations to continue to play a relevant role in the study of the inner molecular workings driving DNA replication (and DNA metabolism, in general). Many of these challenges are currently the subject of intense research. One major limitation of some *in vitro* single-molecule technologies is their low throughput, which implies that acquisition of statistically significant results is very time consuming. Recent developments in acoustic force spectroscopy [165], multiplexing magnetic tweezers [166], [167], [168] and microfluidic systems [169] overcame this issue, at least partially, by allowing researchers to obtain increasingly large sets of high-resolution data. Furthermore, to elucidate the complex dynamics and mechano-chemical processes that govern the operation of a multi-nucleoprotein complex such as the replisome, it will be necessary to interrogate different variables of the system simultaneously. In this regard, the recent development of hybrid methods that combine single-molecule manipulation with fluorescence microscopy [103], [170], [171], [172], [173], [174], [175] will enable to correlate the real-time kinetics of DNA replication to the structural organization and/or to inter- or intramolecular structural changes of the replisome components. Progress in this area will be conditioned by advances in chemical methods that allow efficient fluorescent labeling of proteins without affecting their function. Also, the combination of optical tweezers with precise temperature control systems [101], will help to define the crucial role of temperature on the real-time kinetics and mechano-chemistry of DNA replication. Another drawback of single-molecule manipulation methods is that manipulation is often restricted to a specific spatial coordinate. New optical tweezers set ups with multiple, freely adjustable optical traps will undoubtedly help to overcome this issue [176]. Ultimately, characterization of the real-time kinetics, dynamics and mechano-chemistry of individual replisomes in the context of molecular crowding characteristic of living cells will require transfer single-molecule position and force detection techniques to *in vivo* conditions. To this end, non-invasive approaches can be envisaged that make use of molecular force probes that change state as a function of the force applied to them [177], [178] and/or spectroscopically stable fluorophores that change their emission spectra as a function of mechanical force [179], [180]. As stated above, many of these challenges are currently the subject of significant interdisciplinary research, because ‘the future is now’.

## CRediT authorship contribution statement

**Rebeca Bocanegra:** Writing - original draft, Visualization. **Ismael Plaza G.A.:** Writing - original draft. **Carlos R. Pulido:** Writing - original draft. **Borja Ibarra:** Writing - original draft, Writing - review & editing, Visualization, Funding acquisition.

## Declaration of Competing Interest

The authors declare that they have no known competing financial interests or personal relationships that could have appeared to influence the work reported in this paper.

## References

[1] Watson J.D., Crick F.H.C. (1953). Molecular Structure of Nucleic Acids: A Structure for Deoxyribose Nucleic Acid. Nature.

[2] Bessman M.J., Lehman I.R., Simms E.S., Kornberg A. (1958). Enzymatic synthesis of deoxyribonucleic acid. II. General properties of the reaction. The Journal of biological chemistry..

[3] Lehman I.R., Bessman M.J., Simms E.S., Kornberg A. (1958). Enzymatic synthesis of deoxyribonucleic acid. I. Preparation of substrates and partial purification of an enzyme from Escherichia coli. The Journal of biological chemistry..

[4] Meselson M., Stahl F.W. (1958). The replication of DNA in &lt;em>Escherichia coli&lt;/em&gt. Proc Natl Acad Sci.

[5] Snedeker J., Wooten M., Chen X. (2017). The Inherent Asymmetry of DNA Replication. Annu Rev Cell Dev Biol..

[6] Kornberg AB, T. A. DNA Replication. Freeman, New York 1992.

[7] Burgers P.M.J., Kunkel T.A. (2017). Eukaryotic DNA Replication Fork. Annu Rev Biochem.

[8] Yao N.Y., O'Donnell M.E. (2021). The DNA Replication Machine: Structure and Dynamic Function. Sub-cellular biochemistry..

[9] Dulin D., Lipfert J., Moolman M.C., Dekker N.H. (2013). Studying genomic processes at the single-molecule level: introducing the tools and applications. Nat Rev Genet.

[10] Elting Mary W., Spudich J.A. (2012). Future Challenges in Single-Molecule Fluorescence and Laser Trap Approaches to Studies of Molecular Motors. Dev Cell.

[11] Kapadia N., Reyes-Lamothe R. (2019). A quest for coordination among activities at the replisome. Biochem Soc Trans.

[12] Michaelis J., Muschielok A., Andrecka J., Kügel W., Moffitt J.R. (2009). DNA based molecular motors. Phys Life Rev.

[13] Mohapatra S., Lin C.-T., Feng X.A., Basu A., Ha T. (2020). Single-Molecule Analysis and Engineering of DNA Motors. Chem Rev.

[14] Monachino E., Spenkelink L.M., van Oijen A.M. (2017). Watching cellular machinery in action, one molecule at a time. J Cell Biol..

[15] Mueller S.H., Spenkelink L.M., van Oijen A.M. (2019). When proteins play tag: the dynamic nature of the replisome. Biophys Rev.

[16] Stratmann S.A., van Oijen A.M. (2014). DNA replication at the single-molecule level. Chem Soc Rev.

[17] van Oijen A.M., Loparo J.J. (2010). Single-molecule studies of the replisome. Annu Rev Biophys.

[18] Dulin D., Cui T.J., Cnossen J., Docter M.W., Lipfert J., Dekker N.H. (2015). High Spatiotemporal-Resolution Magnetic Tweezers: Calibration and Applications for DNA Dynamics. Biophys J.

[19] Gardini L., Tempestini A., Pavone F.S., Capitanio M. (2018). High-Speed Optical Tweezers for the Study of Single Molecular Motors. Methods in molecular biology (Clifton, NJ)..

[20] Keller D., Bustamante C. (2000). The mechanochemistry of molecular motors. Biophys J.

[21] Moffitt J.R., Chemla Y.R., Smith S.B., Bustamante C. (2008). Recent advances in optical tweezers. Annu Rev Biochem..

[22] Roy R., Hohng S., Ha T. (2008). A practical guide to single-molecule FRET. Nat Methods..

[23] Sarkar SK, Bumb A, Mills M, Neuman KC. SnapShot: single-molecule fluorescence. Cell. 2013;153(6):1408-.e1. Epub 2013/06/12. doi: 10.1016/j.cell.2013.05.024. PubMed PMID: 23746850. 10.1016/j.cell.2013.05.024 23746850

[24] Sasmal D.K., Pulido L.E., Kasal S., Huang J. (2016). Single-molecule fluorescence resonance energy transfer in molecular biology. Nanoscale..

[25] Seol Y, Neuman KC. SnapShot: force spectroscopy and single-molecule manipulation. Cell. 2013;153(5):1168-.e1. Epub 2013/05/28. doi: 10.1016/j.cell.2013.04.047. 10.1016/j.cell.2013.04.047 23706746

[26] Shashkova S, Leake MC. Single-molecule fluorescence microscopy review: shedding new light on old problems. Biosci Rep. 2017;37(4):BSR20170031. doi: 10.1042/BSR20170031 10.1042/BSR20170031 PMC5520217 28694303

[27] Doublié S, Tabor S, Long AM, Richardson CC, Ellenberger T. Crystal structure of a bacteriophage T7 DNA replication complex at 2.2 A resolution. Nature. 1998;391(6664):251-8. Epub 1998/01/24. doi: 10.1038/34593. 10.1038/34593 9440688

[28] Joyce C.M., Steitz T.A. (1994). Function and structure relationships in DNA polymerases. Annu Rev Biochem..

[29] Steitz TA. A mechanism for all polymerases. Nature. 1998;391(6664):231-2. Epub 1998/01/24. doi: 10.1038/34542. 10.1038/34542 9440683

[30] Berman A.J., Kamtekar S., Goodman J.L., Lázaro J.M., de Vega M., Blanco L. (2007). Structures of phi29 DNA polymerase complexed with substrate: the mechanism of translocation in B-family polymerases. The EMBO journal..

[31] Golosov AA, Warren JJ, Beese LS, Karplus M. The mechanism of the translocation step in DNA replication by DNA polymerase I: a computer simulation analysis. Structure (London, England : 1993). 2010;18(1):83-93. Epub 2010/02/16. doi: 10.1016/j.str.2009.10.014. 10.1016/j.str.2009.10.014 PMC3325112 20152155

[32] Dahl JM, Mai AH, Cherf GM, Jetha NN, Garalde DR, Marziali A, et al. Direct observation of translocation in individual DNA polymerase complexes. The Journal of biological chemistry. 2012;287(16):13407-21. Epub 2012/03/02. doi: 10.1074/jbc.M111.338418. 10.1074/jbc.M111.338418 PMC3339981 22378784

[33] Lieberman K.R., Cherf G.M., Doody M.J., Olasagasti F., Kolodji Y., Akeson M. (2010). Processive Replication of Single DNA Molecules in a Nanopore Catalyzed by phi29 DNA Polymerase. J Am Chem Soc.

[34] Lieberman K.R., Dahl J.M., Mai A.H., Akeson M., Wang H. (2012). Dynamics of the Translocation Step Measured in Individual DNA Polymerase Complexes. J Am Chem Soc.

[35] Lieberman K.R., Dahl J.M., Mai A.H., Cox A., Akeson M., Wang H. (2013). Kinetic Mechanism of Translocation and dNTP Binding in Individual DNA Polymerase Complexes. J Am Chem Soc.

[36] Olasagasti F., Lieberman K.R., Benner S., Cherf G.M., Dahl J.M., Deamer D.W. (2010). Replication of individual DNA molecules under electronic control using a protein nanopore. Nat Nanotechnol.

[37] Morin JA, Cao FJ, Lázaro JM, Arias-Gonzalez JR, Valpuesta JM, Carrascosa JL, et al. Mechano-chemical kinetics of DNA replication: identification of the translocation step of a replicative DNA polymerase. Nucleic acids research. 2015;43(7):3643-52. Epub 2015/03/25. doi: 10.1093/nar/gkv204. 10.1093/nar/gkv204 PMC4402526 25800740

[38] Guajardo R, Sousa R. A model for the mechanism of polymerase translocation. Journal of molecular biology. 1997;265(1):8-19. Epub 1997/01/10. doi: 10.1006/jmbi.1996.0707. PubMed PMID: 8995520. 10.1006/jmbi.1996.0707 8995520

[39] Wang M., Li R., Shu B., Jing X., Ye H.-Q., Gong P. (2020). Stringent control of the RNA-dependent RNA polymerase translocation revealed by multiple intermediate structures. Nat Commun.

[40] Kunkel T.A., Bebenek K. (2000). DNA Replication Fidelity. Annu Rev Biochem.

[41] Berezhna SY, Gill JP, Lamichhane R, Millar DP. Single-molecule Förster resonance energy transfer reveals an innate fidelity checkpoint in DNA polymerase I. J Am Chem Soc. 2012;134(27):11261-8. Epub 2012/06/02. doi: 10.1021/ja3038273. 10.1021/ja3038273 PMC3448555 22650319

[42] Evans G.W., Hohlbein J., Craggs T., Aigrain L., Kapanidis A.N. (2015). Real-time single-molecule studies of the motions of DNA polymerase fingers illuminate DNA synthesis mechanisms. Nucleic Acids Res.

[43] Hohlbein J., Aigrain L., Craggs T.D., Bermek O., Potapova O., Shoolizadeh P. (2013). Conformational landscapes of DNA polymerase I and mutator derivatives establish fidelity checkpoints for nucleotide insertion. Nat Commun.

[44] Joyce C.M., Potapova O., DeLucia A.M., Huang X., Basu V.P., Grindley N.D.F. (2008). Fingers-closing and other rapid conformational changes in DNA polymerase I (Klenow fragment) and their role in nucleotide selectivity. Biochemistry.

[45] Markiewicz R.P., Vrtis K.B., Rueda D., Romano L.J. (2012). Single-molecule microscopy reveals new insights into nucleotide selection by DNA polymerase I. Nucleic Acids Res.

[46] Rothwell PJ, Allen WJ, Sisamakis E, Kalinin S, Felekyan S, Widengren J, et al. dNTP-dependent conformational transitions in the fingers subdomain of Klentaq1 DNA polymerase: insights into the role of the “nucleotide-binding” state. The Journal of biological chemistry. 2013;288(19):13575-91. Epub 2013/03/26. doi: 10.1074/jbc.M112.432690. 10.1074/jbc.M112.432690 PMC3650393 23525110

[47] Rothwell P.J., Mitaksov V., Waksman G. (2005). Motions of the fingers subdomain of klentaq1 are fast and not rate limiting: implications for the molecular basis of fidelity in DNA polymerases. Mol Cell.

[48] Santoso Y, Joyce CM, Potapova O, Le Reste L, Hohlbein J, Torella JP, et al. Conformational transitions in DNA polymerase I revealed by single-molecule FRET. Proceedings of the National Academy of Sciences of the United States of America. 2010;107(2):715-20. Epub 2010/01/19. doi: 10.1073/pnas.0910909107. 10.1073/pnas.0910909107 PMC2818957 20080740

[49] Christian T.D., Romano L.J., Rueda D. (2009). Single-molecule measurements of synthesis by DNA polymerase with base-pair resolution. Proc Natl Acad Sci.

[50] Gahlon H.L., Walker A.R., Cisneros G.A., Lamers M.H., Rueda D.S. (2018). Reduced structural flexibility for an exonuclease deficient DNA polymerase III mutant. PCCP.

[51] Lamichhane R, Berezhna SY, Gill JP, Van der Schans E, Millar DP. Dynamics of site switching in DNA polymerase. J Am Chem Soc. 2013;135(12):4735-42. Epub 2013/02/16. doi: 10.1021/ja311641b. 10.1021/ja311641b PMC3706079 23409810

[52] Maxwell B.A., Suo Z. (2013). Single-molecule investigation of substrate binding kinetics and protein conformational dynamics of a B-family replicative DNA polymerase. J Biol Chem.

[53] Park J, Jergic S, Jeon Y, Cho WK, Lee R, Dixon NE, et al. Dynamics of Proofreading by the E. coli Pol III Replicase. Cell chemical biology. 2018;25(1):57-66.e4. Epub 2017/11/07. doi: 10.1016/j.chembiol.2017.09.008. PubMed PMID: 29104063. 10.1016/j.chembiol.2017.09.008 29104063

[54] Hoekstra T.P., Depken M., Lin S.-N., Cabanas-Danés J., Gross P., Dame R.T. (2017). Switching between Exonucleolysis and Replication by T7 DNA Polymerase Ensures High Fidelity. Biophys J.

[55] Ibarra B., Chemla Y.R., Plyasunov S., Smith S.B., Lázaro J.M., Salas M. (2009). Proofreading dynamics of a processive DNA polymerase. The EMBO journal..

[56] Maier B, Bensimon D, Croquette V. Replication by a single DNA polymerase of a stretched single-stranded DNA. Proceedings of the National Academy of Sciences of the United States of America. 2000;97(22):12002-7. Epub 2000/10/26. doi: 10.1073/pnas.97.22.12002. 10.1073/pnas.97.22.12002 PMC17284 11050232

[57] Naufer M.N., Murison D.A., Rouzina I., Beuning P.J., Williams M.C. (2017). Single-molecule mechanochemical characterization of E. coli pol III core catalytic activity. Protein Sci..

[58] Wuite G.J.L., Smith S.B., Young M., Keller D., Bustamante C. (2000). Single-molecule studies of the effect of template tension on T7 DNA polymerase activity. Nature.

[59] Canceill D, Viguera E, Ehrlich SD. Replication slippage of different DNA polymerases is inversely related to their strand displacement efficiency. The Journal of biological chemistry. 1999;274(39):27481-90. Epub 1999/09/17. doi: 10.1074/jbc.274.39.27481. 10.1074/jbc.274.39.27481 10488082

[60] Manosas M, Spiering MM, Ding F, Bensimon D, Allemand JF, Benkovic SJ, et al. Mechanism of strand displacement synthesis by DNA replicative polymerases. Nucleic acids research. 2012;40(13):6174-86. Epub 2012/03/22. doi: 10.1093/nar/gks253. 10.1093/nar/gks253 PMC3401438 22434889

[61] Morin J.A., Cao F.J., Lazaro J.M., Arias-Gonzalez J.R., Valpuesta J.M., Carrascosa J.L. (2012). Active DNA unwinding dynamics during processive DNA replication. Proc Natl Acad Sci.

[62] Morin JA, Cao FJ, Valpuesta JM, Carrascosa JL, Salas M, Ibarra B. Manipulation of single polymerase-DNA complexes: a mechanical view of DNA unwinding during replication. Cell cycle (Georgetown, Tex). 2012;11(16):2967-8. Epub 2012/08/09. doi: 10.4161/cc.21389. 10.4161/cc.21389 PMC3442898 22871727

[63] Schwartz JJ, Quake SR. Single molecule measurement of the “speed limit” of DNA polymerase. Proceedings of the National Academy of Sciences of the United States of America. 2009;106(48):20294-9. Epub 2009/11/13. doi: 10.1073/pnas.0907404106. 10.1073/pnas.0907404106 PMC2787106 19906998

[64] Balakrishnan L, Bambara RA. Okazaki fragment metabolism. Cold Spring Harbor perspectives in biology. 2013;5(2). Epub 2013/02/05. doi: 10.1101/cshperspect.a010173. 10.1101/cshperspect.a010173 PMC3552508 23378587

[65] Uhler J.P., Falkenberg M. (2015). Primer removal during mammalian mitochondrial DNA replication. DNA Repair.

[66] Manosas M., Spiering M.M., Ding F., Croquette V., Benkovic S.J. (2012). Collaborative coupling between polymerase and helicase for leading-strand synthesis. Nucleic Acids Res.

[67] Stano N.M., Jeong Y.-J., Donmez I., Tummalapalli P., Levin M.K., Patel S.S. (2005). DNA synthesis provides the driving force to accelerate DNA unwinding by a helicase. Nature.

[68] Medagli B., Onesti S. (2013). Structure and mechanism of hexameric helicases. Adv Exp Med Biol.

[69] Patel S.S., Picha K.M. (2000). Structure and function of hexameric helicases. Annu Rev Biochem..

[70] O'Donnell ME, Li H. The ring-shaped hexameric helicases that function at DNA replication forks. Nature structural & molecular biology. 2018;25(2):122-30. Epub 2018/01/31. doi: 10.1038/s41594-018-0024-x. 10.1038/s41594-018-0024-x PMC5876725 29379175

[71] Kose H.B., Larsen N.B., Duxin J.P., Yardimci H. (2019). Dynamics of the Eukaryotic Replicative Helicase at Lagging-Strand Protein Barriers Support the Steric Exclusion Model. Cell Reports..

[72] Yuan Z., Georgescu R., Bai L., Zhang D., Li H., O’Donnell M.E. (2020). DNA unwinding mechanism of a eukaryotic replicative CMG helicase. Nat Commun.

[73] Sun B, Wang MD. Single-molecule perspectives on helicase mechanisms and functions. Critical reviews in biochemistry and molecular biology. 2016;51(1):15-25. Epub 2015/11/06. doi: 10.3109/10409238.2015.1102195. 10.3109/10409238.2015.1102195 26540349

[74] Duzdevich D., Warner M.D., Ticau S., Ivica N.A., Bell S.P., Greene E.C. (2015). The dynamics of eukaryotic replication initiation: origin specificity, licensing, and firing at the single-molecule level. Mol Cell.

[75] Lee S.-J., Syed S., Enemark E.J., Schuck S., Stenlund A., Ha T. (2014). Dynamic look at DNA unwinding by a replicative helicase. Proc Natl Acad Sci.

[76] Rothenberg E., Trakselis M.A., Bell S.D., Ha T. (2007). MCM forked substrate specificity involves dynamic interaction with the 5'-tail. The Journal of biological chemistry..

[77] Ticau S, Friedman LJ, Ivica NA, Gelles J, Bell SP. Single-molecule studies of origin licensing reveal mechanisms ensuring bidirectional helicase loading. Cell. 2015;161(3):513-25. Epub 2015/04/22. doi: 10.1016/j.cell.2015.03.012. 10.1016/j.cell.2015.03.012 PMC4445235 25892223

[78] Douglas ME, Ali FA, Costa A, Diffley JFX. The mechanism of eukaryotic CMG helicase activation. Nature. 2018;555(7695):265-8. Epub 2018/03/01. doi: 10.1038/nature25787. PubMed PMID: 29489749; PubMed Central PMCID: PMCPMC6847044. 10.1038/nature25787 PMC6847044 29489749

[79] Joo S., Chung B.H., Lee M., Ha T.H. (2019). Ring-shaped replicative helicase encircles double-stranded DNA during unwinding. Nucleic Acids Res.

[80] Kaur P, Longley MJ, Pan H, Wang W, Countryman P, Wang H, et al. Single-molecule level structural dynamics of DNA unwinding by human mitochondrial Twinkle helicase. The Journal of biological chemistry. 2020;295(17):5564-76. Epub 2020/03/28. doi: 10.1074/jbc.RA120.012795. 10.1074/jbc.RA120.012795 PMC7186178 32213598

[81] Wasserman MR, Schauer GD, O'Donnell ME, Liu S. Replication Fork Activation Is Enabled by a Single-Stranded DNA Gate in CMG Helicase. Cell. 2019;178(3):600-11.e16. Epub 2019/07/28. doi: 10.1016/j.cell.2019.06.032. 10.1016/j.cell.2019.06.032 PMC6705614 31348887

[82] Yardimci H, Wang X, Loveland AB, Tappin I, Rudner DZ, Hurwitz J, et al. Bypass of a protein barrier by a replicative DNA helicase. Nature. 2012;492(7428):205-9. Epub 2012/12/04. doi: 10.1038/nature11730. 10.1038/nature11730 PMC3521859 23201686

[83] Yeeles J.T.P., Deegan T.D., Janska A., Early A., Diffley J.F.X. (2015). Regulated eukaryotic DNA replication origin firing with purified proteins. Nature.

[84] Burnham D.R., Kose H.B., Hoyle R.B., Yardimci H. (2019). The mechanism of DNA unwinding by the eukaryotic replicative helicase. Nat Commun.

[85] Nandakumar D, Patel SS. Methods to study the coupling between replicative helicase and leading-strand DNA polymerase at the replication fork. Methods (San Diego, Calif). 2016;108:65-78. Epub 2016/05/14. doi: 10.1016/j.ymeth.2016.05.003. 10.1016/j.ymeth.2016.05.003 PMC5035585 27173619

[86] Pandey M., Patel S.S. (2014). Helicase and Polymerase Move Together Close to the Fork Junction and Copy DNA in One-Nucleotide Steps. Cell Reports..

[87] Sun B, Johnson DS, Patel G, Smith BY, Pandey M, Patel SS, et al. ATP-induced helicase slippage reveals highly coordinated subunits. Nature. 2011;478(7367):132-5. Epub 2011/09/20. doi: 10.1038/nature10409. 10.1038/nature10409 PMC3190587 21927003

[88] Syed S, Pandey M, Patel SS, Ha T. Single-molecule fluorescence reveals the unwinding stepping mechanism of replicative helicase. Cell Rep. 2014;6(6):1037-45. Epub 2014/03/19. doi: 10.1016/j.celrep.2014.02.022. PubMed PMID: 24630993; PubMed Central PMCID: PMCPMC3988844. 10.1016/j.celrep.2014.02.022 PMC3988844 24630993

[89] Johnson D.S., Bai L., Smith B.Y., Patel S.S., Wang M.D. (2007). Single-Molecule Studies Reveal Dynamics of DNA Unwinding by the Ring-Shaped T7 Helicase. Cell.

[90] Lionnet T., Spiering M.M., Benkovic S.J., Bensimon D., Croquette V. (2007). Real-time observation of bacteriophage T4 gp41 helicase reveals an unwinding mechanism. Proc Natl Acad Sci.

[91] Manosas M, Xi XG, Bensimon D, Croquette V. Active and passive mechanisms of helicases. Nucleic acids research. 2010;38(16):5518-26. Epub 2010/04/29. doi: 10.1093/nar/gkq273. PubMed PMID: 20423906; PubMed Central PMCID: PMCPMC2938219. 10.1093/nar/gkq273 PMC2938219 20423906

[92] Ribeck N, Kaplan DL, Bruck I, Saleh OA. DnaB helicase activity is modulated by DNA geometry and force. Biophysical journal. 2010;99(7):2170-9. Epub 2010/10/07. doi: 10.1016/j.bpj.2010.07.039. 10.1016/j.bpj.2010.07.039 PMC3042577 20923651

[93] Ribeck N, Saleh OA. DNA unwinding by ring-shaped T4 helicase gp41 is hindered by tension on the occluded strand. PloS one. 2013;8(11):e79237. Epub 2013/11/20. doi: 10.1371/journal.pone.0079237. PubMed PMID: 24250825; PubMed Central PMCID: PMCPMC3826741. 10.1371/journal.pone.0079237 PMC3826741 24250825

[94] Schermerhorn KM, Tanner N, Kelman Z, Gardner AF. High-temperature single-molecule kinetic analysis of thermophilic archaeal MCM helicases. Nucleic acids research. 2016;44(18):8764-71. Epub 2016/07/07. doi: 10.1093/nar/gkw612. 10.1093/nar/gkw612 PMC5062978 27382065

[95] Graham JE, Marians KJ, Kowalczykowski SC. Independent and Stochastic Action of DNA Polymerases in the Replisome. Cell. 2017;169(7):1201-13.e17. Epub 2017/06/18. doi: 10.1016/j.cell.2017.05.041. 10.1016/j.cell.2017.05.041 PMC5548433 28622507

[96] Hamdan SM, Johnson DE, Tanner NA, Lee JB, Qimron U, Tabor S, et al. Dynamic DNA helicase-DNA polymerase interactions assure processive replication fork movement. Molecular cell. 2007;27(4):539-49. Epub 2007/08/21. doi: 10.1016/j.molcel.2007.06.020. 10.1016/j.molcel.2007.06.020 17707227

[97] Kulczyk AW, Akabayov B, Lee SJ, Bostina M, Berkowitz SA, Richardson CC. An interaction between DNA polymerase and helicase is essential for the high processivity of the bacteriophage T7 replisome. The Journal of biological chemistry. 2012;287(46):39050-60. Epub 2012/09/15. doi: 10.1074/jbc.M112.410647. 10.1074/jbc.M112.410647 PMC3493946 22977246

[98] Patel G, Johnson DS, Sun B, Pandey M, Yu X, Egelman EH, et al. A257T linker region mutant of T7 helicase-primase protein is defective in DNA loading and rescued by T7 DNA polymerase. The Journal of biological chemistry. 2011;286(23):20490-9. Epub 2011/04/26. doi: 10.1074/jbc.M110.201657. 10.1074/jbc.M110.201657 PMC3121467 21515672

[99] Patel G., Johnson D.S., Sun B., Pandey M., Yu X., Egelman E.H. (2011). A257T linker region mutant of T7 helicase-primase protein is defective in DNA loading and rescued by T7 DNA polymerase. The Journal of biological chemistry..

[100] Smith SB, Cui Y, Bustamante C. Optical-trap force transducer that operates by direct measurement of light momentum. Methods in enzymology. 2003;361:134-62. Epub 2003/03/11. doi: 10.1016/s0076-6879(03)61009-8. PubMed PMID: 12624910. 10.1016/s0076-6879(03)61009-8 12624910

[101] de Lorenzo S., Ribezzi-Crivellari M., Arias-Gonzalez J.R., Smith S.B., Ritort F. (2015). A Temperature-Jump Optical Trap for Single-Molecule Manipulation. Biophys J.

[102] Camunas-Soler J., Ribezzi-Crivellari M., Ritort F. (2016). Elastic Properties of Nucleic Acids by Single-Molecule Force Spectroscopy. Annu Rev Biophys.

[103] Whitley K.D., Comstock M.J., Chemla Y.R. (2017). High-Resolution “Fleezers”: Dual-Trap Optical Tweezers Combined with Single-Molecule Fluorescence Detection. Methods in molecular biology (Clifton, NJ)..

[104] Suksombat S, Khafizov R, Kozlov AG, Lohman TM, Chemla YR. Structural dynamics of E. coli single-stranded DNA binding protein reveal DNA wrapping and unwrapping pathways. eLife. 2015;4:e08193. doi: 10.7554/eLife.08193 10.7554/eLife.08193 PMC4582245 26305498

[105] Antony E, Lohman TM. Dynamics of E. coli single stranded DNA binding (SSB) protein-DNA complexes. Seminars in cell & developmental biology. 2019;86:102-11. Epub 2018/03/29. doi: 10.1016/j.semcdb.2018.03.017. 10.1016/j.semcdb.2018.03.017 PMC6165710 29588158

[106] Shereda RD, Kozlov AG, Lohman TM, Cox MM, Keck JL. SSB as an organizer/mobilizer of genome maintenance complexes. Critical reviews in biochemistry and molecular biology. 2008;43(5):289-318. Epub 2008/10/22. doi: 10.1080/10409230802341296. 10.1080/10409230802341296 PMC2583361 18937104

[107] Flynn RL, Zou L. Oligonucleotide/oligosaccharide-binding fold proteins: a growing family of genome guardians. Critical reviews in biochemistry and molecular biology. 2010;45(4):266-75. Epub 2010/06/03. doi: 10.3109/10409238.2010.488216. 10.3109/10409238.2010.488216 PMC2906097 20515430

[108] Lohman T.M., Bujalowski W., Overman L.B.E. (1988). coli single strand binding protein: a new look at helix-destabilizing proteins. Trends Biochem Sci.

[109] Antony E, Kozlov AG, Nguyen B, Lohman TM. Plasmodium falciparum SSB tetramer binds single-stranded DNA only in a fully wrapped mode. Journal of molecular biology. 2012;420(4-5):284-95. Epub 2012/05/01. doi: 10.1016/j.jmb.2012.04.022. 10.1016/j.jmb.2012.04.022 PMC3894689 22543238

[110] Gibb B, Ye LF, Gergoudis SC, Kwon Y, Niu H, Sung P, et al. Concentration-dependent exchange of replication protein A on single-stranded DNA revealed by single-molecule imaging. PloS one. 2014;9(2):e87922. Epub 2014/02/06. doi: 10.1371/journal.pone.0087922. 10.1371/journal.pone.0087922 PMC3912175 24498402

[111] Hatch K., Danilowicz C., Coljee V., Prentiss M. (2007). Direct measurements of the stabilization of single-stranded DNA under tension by single-stranded binding proteins. Phys Rev E: Stat Nonlinear Soft Matter Phys.

[112] Hatch K, Danilowicz C, Coljee V, Prentiss M. Measurement of the salt-dependent stabilization of partially open DNA by Escherichia coli SSB protein. Nucleic acids research. 2008;36(1):294-9. Epub 2007/11/23. doi: 10.1093/nar/gkm1014. 10.1093/nar/gkm1014 PMC2248746 18032436

[113] Lee W., Gillies J.P., Jose D., Israels B.A., von Hippel P.H., Marcus A.H. (2016). Single-molecule FRET studies of the cooperative and non-cooperative binding kinetics of the bacteriophage T4 single-stranded DNA binding protein (gp32) to ssDNA lattices at replication fork junctions. Nucleic Acids Res.

[114] Morten MJ, Peregrina JR, Figueira-Gonzalez M, Ackermann K, Bode BE, White MF, et al. Binding dynamics of a monomeric SSB protein to DNA: a single-molecule multi-process approach. Nucleic acids research. 2015;43(22):10907-24. Epub 2015/11/19. doi: 10.1093/nar/gkv1225. PubMed PMID: 26578575; PubMed Central PMCID: PMCPMC4678828. 10.1093/nar/gkv1225 PMC4678828 26578575

[115] Pant K, Karpel RL, Rouzina I, Williams MC. Mechanical measurement of single-molecule binding rates: kinetics of DNA helix-destabilization by T4 gene 32 protein. Journal of molecular biology. 2004;336(4):851-70. Epub 2004/04/21. doi: 10.1016/j.jmb.2003.12.025. 10.1016/j.jmb.2003.12.025 15095865

[116] Pant K, Karpel RL, Rouzina I, Williams MC. Salt dependent binding of T4 gene 32 protein to single and double-stranded DNA: single molecule force spectroscopy measurements. Journal of molecular biology. 2005;349(2):317-30. Epub 2005/05/14. doi: 10.1016/j.jmb.2005.03.065. PubMed PMID: 15890198. 10.1016/j.jmb.2005.03.065 15890198

[117] Shokri L, Marintcheva B, Eldib M, Hanke A, Rouzina I, Williams MC. Kinetics and thermodynamics of salt-dependent T7 gene 2.5 protein binding to single- and double-stranded DNA. Nucleic acids research. 2008;36(17):5668-77. Epub 2008/09/06. doi: 10.1093/nar/gkn551. PubMed PMID: 18772224; PubMed Central PMCID: PMCPMC2553585. 10.1093/nar/gkn551 PMC2553585 18772224

[118] Shokri L, Marintcheva B, Richardson CC, Rouzina I, Williams MC. Single molecule force spectroscopy of salt-dependent bacteriophage T7 gene 2.5 protein binding to single-stranded DNA. The Journal of biological chemistry. 2006;281(50):38689-96. E 10.1074/jbc.M608460200 17050544

[119] Tan HY, Wilczek LA, Pottinger S, Manosas M, Yu C, Nguyenduc T, et al. The intrinsically disordered linker of E. coli SSB is critical for the release from single-stranded DNA. Protein Sci. 2017;26(4):700-17. Epub 2017/01/13. doi: 10.1002/pro.3115. 10.1002/pro.3115 PMC5368059 28078720

[120] Zhang W., Lü X., Zhang W., Shen J. (2011). EMSA and Single-Molecule Force Spectroscopy Study of Interactions between Bacillus subtilis Single-Stranded DNA-Binding Protein and Single-Stranded DNA. Langmuir.

[121] Lohman TM, Ferrari ME. Escherichia coli single-stranded DNA-binding protein: multiple DNA-binding modes and cooperativities. Annu Rev Biochem. 1994;63:527-70. Epub 1994/01/01. doi: 10.1146/annurev.bi.63.070194.002523. 10.1146/annurev.bi.63.070194.002523 7979247

[122] Roy R., Kozlov A.G., Lohman T.M., Ha T. (2007). Dynamic Structural Rearrangements Between DNA Binding Modes of E. coli SSB Protein. J Mol Biol.

[123] Roy R., Kozlov A.G., Lohman T.M., Ha T. (2009). SSB protein diffusion on single-stranded DNA stimulates RecA filament formation. Nature.

[124] Zhou R, Kozlov AG, Roy R, Zhang J, Korolev S, Lohman TM, et al. SSB functions as a sliding platform that migrates on DNA via reptation. Cell. 2011;146(2):222-32. Epub 2011/07/26. doi: 10.1016/j.cell.2011.06.036. 10.1016/j.cell.2011.06.036 PMC3155616 21784244

[125] Nguyen B, Sokoloski J, Galletto R, Elson EL, Wold MS, Lohman TM. Diffusion of human replication protein A along single-stranded DNA. Journal of molecular biology. 2014;426(19):3246-61. Epub 2014/07/25. doi: 10.1016/j.jmb.2014.07.014. 10.1016/j.jmb.2014.07.014 PMC4150847 25058683

[126] Bell JC, Liu B, Kowalczykowski SC. Imaging and energetics of single SSB-ssDNA molecules reveal intramolecular condensation and insight into RecOR function. eLife. 2015;4:e08646. doi: 10.7554/eLife.08646. 10.7554/eLife.08646 PMC4652220 26381353

[127] Lee KS, Marciel AB, Kozlov AG, Schroeder CM, Lohman TM, Ha T. Ultrafast redistribution of E. coli SSB along long single-stranded DNA via intersegment transfer. Journal of molecular biology. 2014;426(13):2413-21. Epub 2014/05/06. doi: 10.1016/j.jmb.2014.04.023. 10.1016/j.jmb.2014.04.023 PMC4096553 24792418

[128] Spenkelink LM, Lewis JS, Jergic S, Xu ZQ, Robinson A, Dixon NE, et al. Recycling of single-stranded DNA-binding protein by the bacterial replisome. Nucleic acids research. 2019;47(8):4111-23. Epub 2019/02/16. doi: 10.1093/nar/gkz090. 10.1093/nar/gkz090 PMC6486552 30767010

[129] Cerrón F, de Lorenzo S, Lemishko KM, Ciesielski GL, Kaguni LS, Cao FJ, et al. Replicative DNA polymerases promote active displacement of SSB proteins during lagging strand synthesis. Nucleic acids research. 2019;47(11):5723-34. Epub 2019/04/11. doi: 10.1093/nar/gkz249. 10.1093/nar/gkz249 PMC6582349 30968132

[130] Georgescu RE, Yao N, Indiani C, Yurieva O, O'Donnell ME. Replisome mechanics: lagging strand events that influence speed and processivity. Nucleic acids research. 2014;42(10):6497-510. Epub 2014/05/16. doi: 10.1093/nar/gku257. 10.1093/nar/gku257 PMC4041431 24829446

[131] Kose H.B., Xie S., Cameron G., Strycharska M.S., Yardimci H. (2020). Duplex DNA engagement and RPA oppositely regulate the DNA-unwinding rate of CMG helicase. Nat Commun.

[132] Morin J.A., Cerrón F., Jarillo J., Beltran-Heredia E., Ciesielski G.L., Arias-Gonzalez J.R. (2017). DNA synthesis determines the binding mode of the human mitochondrial single-stranded DNA-binding protein. Nucleic Acids Res.

[133] Sarkar R., Rybenkov V.V. (2016). A Guide to Magnetic Tweezers and Their Applications. Frontiers. Physics..

[134] Manosas M., Meglio A., Spiering M.M., Ding F., Benkovic S.J., Barre F.X. (2010). Magnetic tweezers for the study of DNA tracking motors. Methods Enzymol.

[135] Manosas M., Spiering M.M., Zhuang Z., Benkovic S.J., Croquette V. (2009). Coupling DNA unwinding activity with primer synthesis in the bacteriophage T4 primosome. Nat Chem Biol.

[136] Smiley RD, Zhuang Z, Benkovic SJ, Hammes GG. Single-molecule investigation of the T4 bacteriophage DNA polymerase holoenzyme: multiple pathways of holoenzyme formation. Biochemistry. 2006;45(26):7990-7. Epub 2006/06/28. doi: 10.1021/bi0603322. PubMed PMID: 16800624; PubMed Central PMCID: PMCPMC2516556. 10.1021/bi0603322 PMC2516556 16800624

[137] Phelps C., Lee W., Jose D., von Hippel P.H., Marcus A.H. (2013). Single-molecule FRET and linear dichroism studies of DNA breathing and helicase binding at replication fork junctions. Proc Natl Acad Sci.

[138] Zhang Z., Spiering M.M., Trakselis M.A., Ishmael F.T., Xi J., Benkovic S.J. (2005). Assembly of the bacteriophage T4 primosome: single-molecule and ensemble studies. PNAS.

[139] Jose D, Weitzel SE, Jing D, von Hippel PH. Assembly and subunit stoichiometry of the functional helicase-primase (primosome) complex of bacteriophage T4. Proceedings of the National Academy of Sciences. 2012;109(34):13596. doi: 10.1073/pnas.1210040109. 10.1073/pnas.1210040109 PMC3427118 22869700

[140] Lee W., Jose D., Phelps C., Marcus A.H., von Hippel P.H. (2013). A Single-Molecule View of the Assembly Pathway, Subunit Stoichiometry, and Unwinding Activity of the Bacteriophage T4 Primosome (helicase–primase) Complex. Biochemistry.

[141] Xi J., Zhang Z., Zhuang Z., Yang J., Spiering M.M., Hammes G.G. (2005). Interaction between the T4 helicase loading protein (gp59) and the DNA polymerase (gp43): unlocking of the gp59-gp43-DNA complex to initiate assembly of a fully functional replisome. Biochemistry.

[142] Park K., Debyser Z., Tabor S., Richardson C.C., Griffith J.D. (1998). Formation of a DNA Loop at the Replication Fork Generated by Bacteriophage T7 Replication Proteins *. J Biol Chem.

[143] Sinha N.K., Morris C.F., Alberts B.M. (1980). Efficient in vitro replication of double-stranded DNA templates by a purified T4 bacteriophage replication system. J Biol Chem.

[144] Hamdan S.M., Loparo J.J., Takahashi M., Richardson C.C., van Oijen A.M. (2009). Dynamics of DNA replication loops reveal temporal control of lagging-strand synthesis. Nature.

[145] Pandey M., Syed S., Donmez I., Patel G., Ha T., Patel S.S. (2009). Coordinating DNA replication by means of priming loop and differential synthesis rate. Nature.

[146] Loparo J.J., Kulczyk A.W., Richardson C.C., van Oijen A.M. (2011). Simultaneous single-molecule measurements of phage T7 replisome composition and function reveal the mechanism of polymerase exchange. Proc Natl Acad Sci.

[147] Geertsema H.J., Kulczyk A.W., Richardson C.C., van Oijen A.M. (2014). Single-molecule studies of polymerase dynamics and stoichiometry at the bacteriophage T7 replication machinery. Proc Natl Acad Sci.

[148] Duderstadt K.E., Geertsema H.J., Stratmann S.A., Punter C.M., Kulczyk A.W., Richardson C.C. (2016). Simultaneous Real-Time Imaging of Leading and Lagging Strand Synthesis Reveals the Coordination Dynamics of Single Replisomes. Mol Cell.

[149] McInerney P, Johnson A, Katz F, O'Donnell M. Characterization of a triple DNA polymerase replisome. Molecular cell. 2007;27(4):527-38. Epub 2007/08/21. doi: 10.1016/j.molcel.2007.06.019. PubMed PMID: 17707226. 10.1016/j.molcel.2007.06.019 17707226

[150] Reyes-Lamothe R., Sherratt D.J., Leake M.C. (2010). Stoichiometry and architecture of active DNA replication machinery in Escherichia coli. Science.

[151] Georgescu RE, Kurth I, O'Donnell ME. Single-molecule studies reveal the function of a third polymerase in the replisome. Nature structural & molecular biology. 2011;19(1):113-6. Epub 2011/12/14. doi: 10.1038/nsmb.2179. 10.1038/nsmb.2179 PMC3721970 22157955

[152] Beattie TR, Kapadia N, Nicolas E, Uphoff S, Wollman AJ, Leake MC, et al. Frequent exchange of the DNA polymerase during bacterial chromosome replication. Elife. 2017;6. Epub 2017/04/01. doi: 10.7554/eLife.21763. 10.7554/eLife.21763 PMC5403216 28362256

[153] Lewis JS, Spenkelink LM, Jergic S, Wood EA, Monachino E, Horan NP, et al. Single-molecule visualization of fast polymerase turnover in the bacterial replisome. Elife. 2017;6. Epub 2017/04/23. doi: 10.7554/eLife.23932. 10.7554/eLife.23932 PMC5419744 28432790

[154] Yang J, Zhuang Z, Roccasecca RM, Trakselis MA, Benkovic SJ. The dynamic processivity of the T4 DNA polymerase during replication. Proceedings of the National Academy of Sciences of the United States of America. 2004;101(22):8289. doi: 10.1073/pnas.0402625101. 10.1073/pnas.0402625101 PMC420387 15148377

[155] Indiani C, Langston LD, Yurieva O, Goodman MF, O'Donnell M. Translesion DNA polymerases remodel the replisome and alter the speed of the replicative helicase. Proceedings of the National Academy of Sciences of the United States of America. 2009;106(15):6031-8. Epub 2009/03/13. doi: 10.1073/pnas.0901403106. 10.1073/pnas.0901403106 PMC2654394 19279203

[156] Indiani C, McInerney P, Georgescu R, Goodman MF, O'Donnell M. A sliding-clamp toolbelt binds high- and low-fidelity DNA polymerases simultaneously. Molecular cell. 2005;19(6):805-15. Epub 2005/09/20. doi: 10.1016/j.molcel.2005.08.011. 10.1016/j.molcel.2005.08.011 16168375

[157] Kath JE, Jergic S, Heltzel JM, Jacob DT, Dixon NE, Sutton MD, et al. Polymerase exchange on single DNA molecules reveals processivity clamp control of translesion synthesis. Proceedings of the National Academy of Sciences of the United States of America. 2014;111(21):7647-52. Epub 2014/05/16. doi: 10.1073/pnas.1321076111. 10.1073/pnas.1321076111 PMC4040570 24825884

[158] Tanner NA, Tolun G, Loparo JJ, Jergic S, Griffith JD, Dixon NE, et al. E. coli DNA replication in the absence of free β clamps. The EMBO journal. 2011;30(9):1830-40. Epub 2011/03/29. doi: 10.1038/emboj.2011.84. 10.1038/emboj.2011.84 PMC3101994 21441898

[159] Åberg C, Duderstadt KE, van Oijen AM. Stability versus exchange: a paradox in DNA replication. Nucleic acids research. 2016;44(10):4846-54. Epub 2016/04/27. doi: 10.1093/nar/gkw296. 10.1093/nar/gkw296 PMC4889951 27112565

[160] Lee J.B., Hite R.K., Hamdan S.M., Xie X.S., Richardson C.C., van Oijen A.M. (2006). DNA primase acts as a molecular brake in DNA replication. Nature.

[161] Yao N.Y., Georgescu R.E., Finkelstein J., O'Donnell M.E. (2009). Single-molecule analysis reveals that the lagging strand increases replisome processivity but slows replication fork progression. Proc Natl Acad Sci.

[162] Lewis JS, Spenkelink LM, Schauer GD, Yurieva O, Mueller SH, Natarajan V, et al. Tunability of DNA Polymerase Stability during Eukaryotic DNA Replication. Molecular cell. 2020;77(1):17-25.e5. Epub 2019/11/11. doi: 10.1016/j.molcel.2019.10.005. 10.1016/j.molcel.2019.10.005 PMC6943181 31704183

[163] Schauer GD, O'Donnell ME. Quality control mechanisms exclude incorrect polymerases from the eukaryotic replication fork. Proceedings of the National Academy of Sciences of the United States of America. 2017;114(4):675-80. Epub 2017/01/11. doi: 10.1073/pnas.1619748114. 10.1073/pnas.1619748114 PMC5278475 28069954

[164] Kapadia N., El-Hajj Z.W., Zheng H., Beattie T.R., Yu A., Reyes-Lamothe R. (2020). Processive Activity of Replicative DNA Polymerases in the Replisome of Live Eukaryotic Cells. Mol Cell.

[165] Sitters G., Kamsma D., Thalhammer G., Ritsch-Marte M., Peterman E.J.G., Wuite G.J.L. (2015). Acoustic force spectroscopy. Nat Methods..

[166] Agarwal R., Duderstadt K.E. (2020). Multiplex flow magnetic tweezers reveal rare enzymatic events with single molecule precision. Nat Commun.

[167] Dulin D, Vilfan ID, Berghuis BA, Hage S, Bamford DH, Poranen MM, et al. Elongation-Competent Pauses Govern the Fidelity of a Viral RNA-Dependent RNA Polymerase. Cell Rep. 2015;10(6):983-92. Epub 2015/02/17. doi: 10.1016/j.celrep.2015.01.031. 10.1016/j.celrep.2015.01.031 25683720

[168] Kriegel F., Vanderlinden W., Nicolaus T., Kardinal A., Lipfert J. (2018). Measuring Single-Molecule Twist and Torque in Multiplexed Magnetic Tweezers. Methods in molecular biology (Clifton. NJ).

[169] Granéli A., Yeykal C.C., Prasad T.K., Greene E.C. (2006). Organized arrays of individual DNA molecules tethered to supported lipid bilayers. Langmuir.

[170] Chuang C.-Y., Zammit M., Whitmore M.L., Comstock M.J. (2019). Combined High-Resolution Optical Tweezers and Multicolor Single-Molecule Fluorescence with an Automated Single-Molecule Assembly Line. The journal of physical chemistry A..

[171] Hashemi Shabestari M., Meijering A.E.C., Roos W.H., Wuite G.J.L., Peterman E.J.G. (2017). Recent Advances in Biological Single-Molecule Applications of Optical Tweezers and Fluorescence Microscopy. Methods Enzymol.

[172] Heller I., Sitters G., Broekmans O.D., Farge G., Menges C., Wende W. (2013). STED nanoscopy combined with optical tweezers reveals protein dynamics on densely covered DNA. Nat Methods..

[173] Lang M.J., Fordyce P.M., Engh A.M., Neuman K.C., Block S.M. (2004). Simultaneous, coincident optical trapping and single-molecule fluorescence. Nat Methods..

[174] Long X., Parks J.W., Stone M.D. (2016). Integrated magnetic tweezers and single-molecule FRET for investigating the mechanical properties of nucleic acid. Methods (San Diego, Calif).

[175] Whitley KD, Comstock MJ, Chemla YR. High-Resolution Optical Tweezers Combined With Single-Molecule Confocal Microscopy. Methods in enzymology. 2017;582:137-69. Epub 2017/01/08. doi: 10.1016/bs.mie.2016.10.036. 10.1016/bs.mie.2016.10.036 PMC5540136 28062033

[176] Heller I., Laurens N., Vorselen D., Broekmans O.D., Biebricher A.S., King G.A. (2017). Versatile Quadruple-Trap Optical Tweezers for Dual DNA Experiments. Methods in molecular biology (Clifton. NJ).

[177] Liu Y., Galior K., Ma V.-P.-Y., Salaita K. (2017). Molecular Tension Probes for Imaging Forces at the Cell Surface. Acc Chem Res.

[178] Wang X., Ha T. (2013). Defining single molecular forces required to activate integrin and notch signaling. Science.

[179] Dufrêne Y.F., Evans E., Engel A., Helenius J., Gaub H.E., Müller D.J. (2011). Five challenges to bringing single-molecule force spectroscopy into living cells. Nat Methods..

[180] Oddershede LB. Force probing of individual molecules inside the living cell is now a reality. Nat Chem Biol. 2012;8(11):879-86. Epub 2012/10/19. doi: 10.1038/nchembio.1082. 10.1038/nchembio.1082 23076067

